# Information-Content-Informed Kendall-tau Correlation Methodology: Interpreting Missing Values as Useful Information

**DOI:** 10.1101/2022.02.24.481854

**Published:** 2025-07-21

**Authors:** Robert M Flight, Praneeth S Bhatt, Hunter NB Moseley

**Affiliations:** 1 Markey Cancer Center, University of Kentucky, Lexington, KY 40536, USA; 2 Department of Molecular & Cellular Biochemistry, University of Kentucky, Lexington, KY 40536, USA; 3 Resource Center for Stable Isotope Resolved Metabolomics, University of Kentucky, Lexington, KY 40536, USA; 4 Department of Electrical and Computer Engineering, University of Kentucky, Lexington, KY 40506; 5 Institute for Biomedical Informatics, University of Kentucky, Lexington, KY 40536, USA; 6 Department of Toxicology and Cancer Biology, University of Kentucky, Lexington, KY 40536, USA

## Abstract

**Background:**

Almost all correlation measures currently available are unable to directly handle missing values. Typically, missing values are either ignored completely by removing them or are imputed and used in the calculation of the correlation coefficient. In either case, the correlation value will be impacted based on a perspective that the missing data represents no useful information. However, missing values occur in real data sets for a variety of reasons. In omics data sets that are derived from analytical measurements, a major reason for missing values is that a specific measurable phenomenon falls below the detection limits of the analytical instrumentation (left-censored values). These missing data are not missing at random, but represent useful information by virtue of their “missingness” at one end of the data distribution.

**Results:**

To include this information due to left-censorship missingness, we propose the information-content-informed Kendall-tau (ICI-Kt) methodology. We show how left-censored missing values can be included within the definition of the Kendall-tau correlation coefficient, and how that inclusion leads to an interpretation of information being added to the correlation. We also implement calculations for additional measures of theoretical maxima and pairwise completeness that add further layers of information interpretation in the methodology. Using both simulated and real data sets from RNA-seq, metabolomics, and lipidomics experiments, we demonstrate that the ICI-Kt methodology allows for the inclusion of left-censored missing data values as interpretable information, enabling both improved determination of outlier samples and improved feature-feature network construction. We provide explicitly parallel implementations in both R and Python that allow fast calculations of all the variables used when applying the ICI-Kt methodology on large numbers of samples.

**Conclusions:**

The ICI-Kt methods are available as an R package and Python module on GitHub at https://github.com/moseleyBioinformaticsLab/ICIKendallTau and https://github.com/moseleyBioinformaticsLab/icikt, respectively.

## Background

Correlation as a measure of the relatedness or similarity of two or more sets of data has a long history, with the mathematical technique being used (and abused) in various scientific fields since its introduction [1,2]. More recently, correlation calculations have become a cornerstone statistical method in the analysis and integration of varied omics’ datasets, especially the big five omics: genomics, transcriptomics, proteomics, metabolomics, and epigenomics [3]. Correlation between biomolecular features (nucleotide variants, RNA transcripts, proteins, metabolites, DNA methylation, etc.) may be used to evaluate the relationship strength between pairs of the features as well as to detect and derive correlative structures between groups of features [4]. Moreover, feature-feature correlations can be used to evaluate a dataset based on expected biochemical correlations, for example higher feature-feature correlations within lipid categories versus between lipid categories [5]. Correlation is a foundational method for generating biomolecular feature-feature interaction networks, like those provided by STRING [6], Genemania [7], and WCGNA [8]. Feature-feature correlation may also be used to inform which features are used for imputation of missing values [9].

Often, the first step in omics level analyses is to examine the sample-sample (dis)similarities in various ways using exploratory data analysis or EDA. This can include the examination of decomposition by principal components analysis (PCA), sample-sample pairwise distances, or sample-sample pairwise correlations to highlight biological and batch groups [10–12], double check the appropriateness of planned analyses [13], and check if any samples should be removed prior to statistical analysis (outlier detection and removal) [14]. Outlier detection, in particular, is often required for successful omics data analysis, as any misstep during the experimentation, sample collection, sample preparation, or analytical measurement of individual samples can inject high error and/or variance into the resulting data set [10–12,14,15].

**Figure 1.**
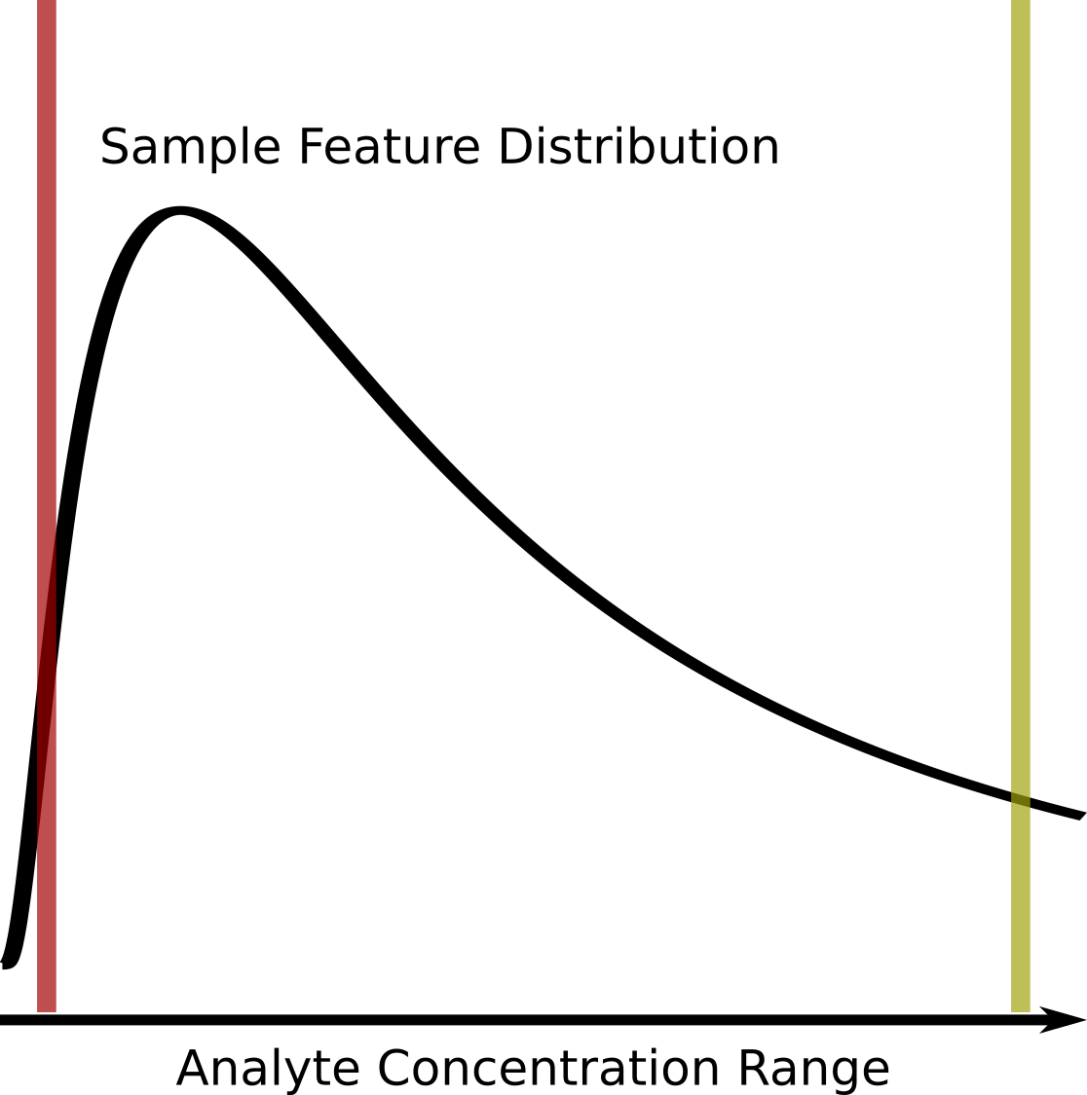
Graphical description of the left-censored data problem. An example density plot of the analyte concentrations for a single experimental sample is shown as a solid black curve. The true analyte concentration range covers the full range of the density distribution, with the minimum on the left (red vertical line), and the maximum on the right (yellow vertical line). Below certain concentrations, shown by the red line, the instrument returns either missing values (NA), zeros, or some other floored values, resulting in a left-censored distribution. Above certain concentrations, highlighted by the yellow line, typically the values returned will be identical (or flagged depending on the instrument). Which analytes will have concentrations below the red detection limit line may vary from sample to sample due to the overall sample composition, as well as natural variances (non-systematic error) within each experimental sample.

All analytical methods, and in particular the analytical methods used in omics where many analytes are being measured simultaneously, suffer from missing measurements. Some analytes will be missing at random because of spurious issues with either the instrument, the particular sample, or sample preparation, but a larger number of missing measurements are left-censored due to analytes being below the effective detection limit of the instrument and the given specific sample preparation procedures utilized, as shown in Figure 1. Some analytical instruments are purposely designed to floor measurements when they occur below a certain signal to noise ratio threshold. Also, imputation of missing measurements in omics samples is an active area of research, which we will not comprehensively cover here beyond to say that it is worthwhile and very necessary in many instances. Imputation methods rely on very similar analytical detection limits between analytical samples. When this condition does not hold, imputation methods have reduced performance and lower interpretive value. For analytical techniques requiring complex sample handling and detection, the variability in the analytical detection level can be quite high. However, when it comes to calculating correlation, there are very few methods that explicitly account for left-censored missing data that we know of. In many cases, missing values are either ignored or imputed to zero (or another value) and then included in the correlation calculation. The two most common approaches for ignoring (i.e. dropping) values is to only use those measurements that are common across all samples (complete) or that are common between two samples being compared (pairwise complete). Both dropping or imputing missing values are likely to cause the calculated sample-sample correlation values to deviate from the real sample-sample correlation values, especially with respect to specific data interpretation perspectives.

Assuming that a majority of missing values are not missing at random, but rather result from left-censored distributions due to the analyte being below the effective detection limit (see Figure 1), we propose that these missing values do in fact encode useful information that can be incorporated into correlation calculations.

To create a correlation measure that is capable of working with missing values, we would not be interested in creating a completely new correlation metric from scratch, but modifying an existing one. Of the three commonly used correlation measures, Pearson, Spearman, and Kendall-tau, Spearman and Kendall-tau seem most appropriate for modification as they solely use ranks in the calculation of their coefficients. Modifying Pearson would either involve imputing new values, or finding a way to calculate the covariances with missingness included. While Spearman uses ranks, many of the modifications for handling identical ranks and ties do not seem amenable to working with missing values. In contrast, Kendall-tau’s use of concordant and discordant pair counts seems most amenable to the creation of new definitions that incorporate missingness while still working within the original definition of the correlation coefficient, as shown in the Implementation section below.

In this work, we propose new definitions of concordant and discordant rank pairs that include missing values, as well as methods for incorporating missing values into the number of tied values for the equivalent of the modified Kendall-tau coefficient, the information-content-informed Kendall-tau (ICI-Kt) method. The implementation of the basic calculation of ICI-Kt involves the replacement of missing values with a value lower than the observed values (technically simple imputation), with subsequent calculation of the Kendall-tau-b statistic, as a majority of missing values are the result of left-censorship, they still provide an interpretation from an information-content perspective, which we demonstrate with the equations below. We also developed a statistical test for determining if the cause for missingness is likely left-censorship. With this statistical test, we demonstrate that left-censorship is a real phenomenon in four different experimental data sets. We examine the effect of missing values on various simulated and real datasets, comparing the ICI-Kt methodology with other simpler methods of handling the missing values, namely removing them or imputing them to zero. Given the detrimental effects of including outlier samples in differential analyses, we also evaluate the ability of the ICI-Kt methodology to capture sample-sample pairwise similarities and the determination of outlier samples prior to differential analyses. Finally, we examine how feature-feature networks derived from various feature-feature correlations change within annotations depending on the correlation method used.

All of the code used for this manuscript is available on zenodo [16].

### Implementation

#### Additional Definitions of Concordant and Discordant Pairs to Include Missingness

In the simplest form, the Kendall-tau correlation can be defined as:

$$\tau_{a}=\frac{n_{concordant}-n_{discordant}}{n_{concordant}+n_{discordant}}$$

where $n_{concordant}$ is the the number of concordant pairs and $n_{discordant}$ is the number of discordant pairs. In this case, a pair are any two x-y points, $x_{i}$, $y_{i}$ and $x_{j}$, $y_{j}$, with i≠j, composed from two joint random variables X and Y, where $x_{i}$ represents the *ith value* in X and $y_{i}$ represents the *ith value* in Y. In an omics context, X and Y can represent feature vectors for two experimental samples or two specific features across a set of samples. A concordant pair has the following classical definition:

- $x_{i}>x_{j}$ and $y_{i}>y_{j}$
- $x_{i}<x_{j}$ and $y_{i}<y_{j}$

A discordant pair has the following classical definition [17]:

- $x_{i}>x_{j}$ and $y_{i}<y_{j}$
- $x_{i}<x_{j}$ and $y_{i}>y_{j}$

We can expand the concordant and discordant pair definitions to include missing values (e.g. NA in R). Here ! x indicates x=NA and ! y indicates y=NA, and & is synonymous with “and”. The information-content-informed concordant pair definitions are then:

- $x_{i}>x_{j}$ and $y_{i}>y_{j}$
- $x_{i}<x_{j}$ and $y_{i}<y_{j}$
- $x_{i}>x_{j}$ and $y_{i}\text{\&}!y_{j}$
- $x_{i}<x_{j}$ and $!y_{i}\text{\&}y_{j}$
- $x_{i}\text{\&}!x_{j}$ and $y_{i}>y_{j}$
- $!x_{i}\text{\&}x_{j}$ and $y_{i}<y_{j}$
- $x_{i}\text{\&}!x_{j}$ and $y_{i}\text{\&}!y_{j}$
- $!x_{i}\text{\&}x_{j}$ and $!y_{i}\text{\&}y_{j}$

The information content informed discordant pair definitions are then:

- $x_{i}>x_{j}$ and $y_{i}<y_{j}$
- $x_{i}<x_{j}$ and $y_{i}>y_{j}$
- $x_{i}<x_{j}$ and $!y_{i}\text{\&}y_{j}$
- $x_{i}<x_{j}$ and $y_{i}\text{\&}!y_{j}$
- $x_{i}\text{\&}!x_{j}$ and $y_{i}<y_{j}$
- $!x_{i}\text{\&}x_{j}$ and $y_{i}>y_{j}$
- $x_{i}\text{\&}!x_{j}$ and $!y_{i}\text{\&}y_{j}$
- $!x_{i}\text{\&}x_{j}$ and $y_{i}\text{\&}!y_{j}$

These additional definitions make it possible to interpret a Kendall-tau correlation from the perspective of missing values as additional information, i.e., information-content-informed Kendall-tau (ICI-Kt) methodology.

### Considering Ties

Tied values do not contribute to either of the concordant or discordant pair counts, and the original Kendall-tau formula for the tau-a statistic does not consider the presence of tied values. However, the related tau-b statistic does handle the presence of tied values by adding the tied x and y values to the denominator, and in our special case of missing data, we can add the ties that result from $\left(x_{i}=\text{NA},x_{j}=\text{NA}\right)$ and $\left(y_{i}=\text{NA},y_{j}=\text{NA}\right)$ to $n_{xtie}$ and $n_{ytie}$ [18,19] used in the following equation for tau-b:

$$\tau_{b}=\frac{n_{concordant}-n_{discordant}}{\sqrt{\left(n_{tot}-n_{xtie}\right)\left(n_{tot}-n_{ytie}\right)}}$$

where $n_{tot}$ is the total number of pairs, $n_{xtie}$ are the number of tied values in X, and $n_{ytie}$ are the number of paired values in Y. We can also consider commonly missing values in X and Y specially as well. In the first instance, we remove those x-y points where both values are missing. We refer to this case as the “local” ICI-Kt correlation. It is most appropriate for the comparison of only two experimental samples, where we are concerned with what values are present in the two experimental samples, with the odd case of missingness.

The other case, where we leave ties resulting from points with missing X and Y, we refer to as the “global” ICI-Kt correlation. In this case, every single correlation over multiple comparisons with the same set of features will consider the same number of pair comparisons. This is useful when analyzing and interpreting correlations from a large number of experimental samples, not just two samples.

### Theoretical Maxima

The “global” case also provides an interesting property, whereby we can calculate the theoretical maximum correlation that would be possible to observe given the lowest number of shared missing values. This value can be useful to scale the rest of the observed correlation values across many sample-sample correlations, providing a value that scales an entire dataset appropriately. For any pairwise comparison of two vectors (from experimental samples for example), we can calculate the maximum possible Kendall-tau for that comparison by defining the maximum number of concordant pairs as:

$$tau_{max}=\frac{n_{tot}-n_{xtie}-n_{ytie}+n_{tie}}{\sqrt{\left(n_{tot}-n_{xtie}\right)\left(n_{tot}-n_{ytie}\right)}}$$

where $n_{tie}$ is the number of commonly tied values in both X and Y. Calculating a set of $tau_{max}$ values between all experimental samples, we can take the maximum of the values, and use it to scale all of the obtained Kendall-tau values equally $\left(\tau \times \max\left(tau_{max}\right)\right)$.

### Completeness

As an addition to the correlation value, we also calculate the *completeness* between any two samples. We first measure the number of entries missing in either of the samples being compared, and subtract that from the total number of features in the samples. This defines how many features are potentially *complete* between the two samples. This number, over the total number of features defines the *completeness* fraction.


$$completness=\frac{n_{feat}-N\left(miss_{i}\cup miss_{j}\right)}{n_{feat}}$$

where for any two samples i and j, $n_{feat}$ is the total number of features or entries, and $miss_{i}\cup miss_{j}$ are the features missing in either sample i or j, with N being the total number of missing entries in either sample i or j.

### Implementation Details

We produced an initial reference implementation in R [20], where the various concordant and discordant pair definitions were written as simple logical tests to allow further exploration and validation of faster implementations. During exploration and validation of an early implementation, we discovered that an equivalent calculation was to replace the missing values with a value smaller than all of the values in the two sets being compared. This simplification does not change the interpretation of the effect of left-censored missing values, but it does allow for the direct use of the very fast mergesort based algorithm for calculating $\tau_{b}$ [21].

We re-implemented the mergesort implementation from the *SciPy kendalltau* code [22] in both R (via *Rcpp*) and Python (via cython) to enable fast, easy parallel computations in both languages (using *furrr* and *multiprocessing*, respectively), as well as inclusion of the calculation of $tau_{max}$, which is derived from the same values needed for the calculation of $\tau_{b}$ (see above). The version of the R package used in this manuscript is available on zenodo [23]. In addition to use as an imported Python module, the Python *icikt* module provides a command line interface (CLI) for the ICI-Kt methodology.

### Simulated Data Sets

Simulated feature vectors (analytical samples) are generated by drawing 1000 random values from a log-normal distribution with a mean of 1 and standard deviation (sd) of 0.5 and sorting them in ascending order to create a pair of samples with perfectly positive (1) or negative (−1) correlation values. The negative analytical sample has values sorted in descending order. Missing value indices are generated by randomly sampling up to 499 of the lowest values in each sample. For the negative sample, the indices are also subtracted from 1000 to cause them to be at the lower end of the feature distribution. Finally, missing indices were only inserted into one of the two samples being compared before calculating the correlation. The missing indices are replaced with NA, and then correlations between the analytical samples are calculated.

Another, more realistic, simulated data set is generated by drawing 1000 random values from a log-normal distribution, and adding noise from a normal distribution with a mean of zero and sd of 0.2 to create two statistical samples. Missing values are created in these statistical samples via two methods: 1) by creating intensity cutoffs from 0 to 1.5 in 0.1 increments, values below the cutoff set to missing or zero depending on the calculation; 2) randomly sampling locations in the two-sample matrix ranging from zero to 300 in increments of 50 and setting the indices to missing or zero.

### Brainson EGFR Lung Tumor RNA-Seq Data Set

This RNA-Seq dataset is from null and knock-in EGFR genotype mice mutants. Genotypes include Null (no mutant-EGFR inducible expression), Heterozygous (only one copy of mutant-EGFR), and Homozygous (two copies of mutant EGFR). For each mutant, cells were also grown in different ways and sequenced: (1) 2D plates; (2) 3D organoids; (3) cells sorted and selected using FACS; (4) total tumor without sorting. See Chen et al [24] for the full experimental details. Counts were normalized using *DESeq2* [25] with either the genotype, the growth environment, or the combination of them as the grouping factor. Samples were grouped by their genotype, growth environment, or their combination for the calculation of median abundance across samples.

### Yeast RNA-Seq Data Set

These data were generated and reported as part of two publications evaluating replicate data and differential gene expression using wild-type (WT) and *Snf2* deletion mutant [14,26]. Summarized gene level counts were obtained from a GitHub project maintained by the Barton group [27]. It should be noted that the data are also available from two figshare repositories [28,29]. Counts were normalized using *DESeq2* [25] with the sample genotype of WT or *Snf2* deletion as the experimental factor. Samples were grouped by their genotype for the calculation of median abundance across samples.

The original outliers reported by Gierliński et al. [14] are based on a combination of median correlations, feature outliers, and RNA-seq coverage. Given that we want to compare outliers based on correlation, we re-determined outliers using only correlation calculated by replacing zero counts with missing values and calculating sample-sample pairwise Pearson correlations on the raw feature counts using the genes present in both samples (*pairwise-complete-observations*).

### Adenocarcinoma Recount RNA-Seq Data Set

We downloaded the recount2 TCGA lung cancer data [30], extracted the scaled counts, and trimmed to the Stage I adenocarcinoma samples, and those genes that have a non-zero count in at least one of the samples. Counts were normalized using *DESeq2* [25] with the tissue status as normal or tumor as the experimental factor. Samples were grouped by the tissue status of normal or tumor for the calculation of median abundance across samples.

### Non-Small Cell Lung Cancer Lipidomics Data Set

We previously analyzed a lipidomics data set from non-small-cell lung cancer (NSCLC) [5,31]. Because the assigned peaks represent such a small amount of the overall data, we reprocessed the full dataset to match peaks across samples. We first calculated the assigned peaks standard deviations in parts per million (PPM) to define an appropriate cutoff for matching peaks across samples. A value of 0.5 ppm looked to be wide enough to capture the variances in location of the assigned peaks. For each peak, an interval of 0.5 ppm to either side of the peak was defined. Starting with a random peak, all peaks with an overlapping interval to the starting peak were deemed to be the *same* peak across samples and aggregated. All of the overlapping peaks are removed from the sample peak list, and then the next available peak chosen, and the process repeated until all peaks were accounted for. This process was repeated with four different randomizations of the full peak list to verify that the number of final aggregated peaks was not dependent on the starting peak list order. All iterations showed that the number of final aggregated peaks were within 100 of each other, where we result in close to 34,000 aggregated peaks. Peak intensities in each sample were normalized by the median abundance of all quantified peaks in a sample. For details on the samples, their processing, and generation of assignments, see Mitchell et al [5]. Samples were grouped by the instrument (two different instruments were used to collect the data) and whether they were taken from normal (nearby non-tumor) or tumor tissue for the calculation of median abundance across samples. The previously assigned peaks were matched to the unassigned peaks by finding peaks within a 0.5 ppm cutoff. For lipid category and class voting, we only considered peaks matched that had a single assigned or a single unassigned peak match together, i.e. satisfying a bisection criterion.

### Rat Stamina and Feeding Metabolomics Data Set (Rat)

This metabolomics data was collected as part of a study on muscle selection of fuel source and oxidative capacity in outbred rats [32]. This data is available at the NIH Common Fund’s National Metabolomics Data Repository (NMDR) website, the Metabolomics Workbench, https://www.metabolomicsworkbench.org where it has been assigned Project ID (PR000016). The data can be accessed directly via it’s Project DOI: https://dx.doi.org/10.21228/M86P45 [33]. The mass-spectrometry peak intensities, and sample metadata were taken directly from the MWTAB json file provided by Metabolomics Workbench. Peak intensities were normalized by the non-zero peak abundances in each sample. Samples were grouped by the combination of feeding status (ad lib or calorie restricted) and oxidative capacity (low or high) for the calculation of median abundance across samples.

### Feature Annotations

Pathway annotations for gene transcripts in the RNA-Seq datasets were obtained from Reactome pathways in the *reactome.db* Bioconductor package as well as organism-specific annotation packages [34,35]. Metabolite annotations for the rat stamina metabolites are based on Kyoto Encyclopedia of Genes and Genomes (KEGG) pathways that map from the provided KEGG compound IDs to various KEGG pathways [36]. Compound-pathway annotations were fetched using the *kegg-pull* Python package [37]. Lipid features from the NSCLC lipidomics with assignments are classified into one or more lipid categories using our lipid classifier tool [38]. For each assigned peak, we used voting of the lipid categories and classes from multiple assignments to ascribe a single category and lipid class (where possible).

### Number of Missing Values and Median Rank

For each dataset, the samples were split by experimental factor or treatment of interest (see previous Methods for each dataset). For each feature, the rank of the feature was calculated for each sample where the feature was present, followed by the features median rank, as well as the number of samples the feature was missing from. Grouping the features by the number of missing values, we calculate the median of median ranks, as well as the minimum of median ranks, for the visualization and correlation of the relationship of rank with missing values.

### Binomial Test for Left Censorship

For each dataset, the samples were first split by treatment. In each sample, the median abundance of features present in the sample are calculated. For any feature that is missing in any sample, the values in the present samples are compared to the median value of their corresponding sample. If the value is less than the median value in the sample, that is counted as a success in a binomial test, otherwise it is counted as a failure. The number of successes and failures are aggregated across the treatment splits for calculation in a binomial test, with the null hypothesis as a ratio of 0.5.

### Correlation Methods

For each dataset, we calculated correlations using a variety of methods. In each dataset, there were either zero values or NA values to represent missingness. To start, we replaced all missing values with 0, and then either set them to NA or left them as zero as appropriate. ICI-Kendall-tau with zeros replaced with NA (IK); and then scaled (multiplied) by the completeness metric (IKC). Kendall-tau, with zero replaced with NA, and then using pairwise-complete-observations (Kt). Pearson, with zeros, using pairwise-complete-observations (PB). Pearson, with zeros replaced with NA, using pairwise-complete-observations (PN0). Pearson, with a log(x+1) transform applied, using pairwise-complete-observations (PL1). Pearson, with a log(x) transform, and then setting infinite values to NA values, using pairwise-complete-observations (PL).

### Outlier Detection

For outlier detection, median sample-sample correlations within the sample treatment (genotype, condition, etc) is calculated, and $log\left(1-cor_{median}\right)$ calculated to transform it into a score. Then outliers are determined using *grDevices::boxplot.stats*, which by default are at 1.5X the whiskers in a box-and-whisker plot. As we are interested in only those correlations at the *low* end of correlation (becoming the high end after the subtraction and log-transform), we restrict to only those entries at the *high* end of the score distribution (using *visualizationQualityControl::determine_outliers* [39]). This is equivalent to using the correlation component of the score described by Gierliński et al [14] and setting the other component weights to zero.

Sample-sample correlations are calculated with different sets of features. For each feature, we calculate the number and fraction of samples in a sample treatment that feature had non-zero or non-missing values in, making it possible to filter to a subset of features that are present in a minimum required number of samples.

For each cutoff of non-missing values, we also calculate the median and median-absolute-deviation (MAD) of the sample median correlations, and then the differences of median and MAD between two classes of samples: SNF2 and WT for yeast; normal and tumor for adenocarcinoma; wt and null for “egfrgenotype”; tumor and normal for one instrument for NSCLC; Ad lib high and low for “ratstamina”.

### Feature-Feature Networks and Partitioning

For each of the datasets, we trimmed to features present in 25% or more of any of the sample classes (see above for the various classes of samples in each dataset). Various correlation measures are calculated between all remaining features (Correlation Methods). For any given correlation, we generate the feature-feature network for that dataset-correlation combination. The dataset correlations are transformed to partial correlation. From the distribution of partial correlation values, we consider the fraction of values that make up the 2.5 % of the tail values (for a total of 5%) as the significant partial correlations that can be used as actual edges in the network. The network is then trimmed to only the edges that have a positive weight. For each feature annotation (see Feature Annotations), we calculate three sums of the edge weights.

1. The total sum of edge weights for all edges with features that are annotated to one or more of the annotations (*annotated*).
2. The *within* annotation edge weight sum, where both start and end nodes are annotated to the same annotation.
3. The *outer* annotation edge weight sum, where the start node is part of the annotated set, and the end node is annotated to one of the other annotations.

The partitioning ratio (or q-ratio) is calculated as follows:

$$Q=\sum_{i=1}^{annot}\frac{within_{i}}{annotated}-{\left(\frac{\;outer_{i}}{\;annotated}\right)}^{2}$$

The partitioning ratio was originally designed as a method to determine the optimal clustering of networks, where each member of the network has only a single label [40–43]. In those cases, the partitioning ratio should range between 0 and 1 for non-partitioned and fully partitioned networks, respectively. None of the annotation sources we use have single labels for any features, and therefore the partitioning ratios have a much wider range. However, we expect that better partitioning of the network will be reflected by more positive partitioning ratios.

### Changes In Correlation Due to Changes in Dynamic Range and Imputation

We created a simulated dataset with 1000 features and 100 samples, starting with a sample from a random log-normal distribution with a mean-log value of 1, and standard deviation of 0.5. Uniform noise was added via random normal distribution with a standard deviation of 0.2 to create 100 samples from the base distribution, values were transformed to normal space and then $log_{10}$ applied to have a representation of orders of magnitude and dynamic range. For any maximum level of censoring to be applied, a uniform distribution sample is generated on the range of 0−max with 100 values. Data censoring was applied by taking the minimum observed value for a sample, and adding the censoring value from the uniform distribution. Any values in the sample below the censoring value are set to missing (NA).

Correlations were calculated between samples when no missing values are present (*reference*), and then again after censoring (*trimmed*). Two different correlation methods were used: ICI-Kt; and Pearson correlation. Imputation for Pearson correlation involved replacing all missing values with ½ the lowest observed value in the dataset after censoring. Differences between the reference and trimmed correlations were calculated, as well as the difference in the absolute value of differences of ICI-Kt and Pearson imputed.

### Computing Environment

Most calculations were run on a virtual machine running Ubuntu 18.04.6 LTS, with 80 virtual cores, and 1 TB of RAM. The virtual machine is running on top of a 50 node cluster, each with 4 10-core Intel Xeon CPUs (E7–4820 v4 @ 2.00GHz) with hyperthreading, 3TB of RAM, an 8TB solid-state-drive array, and a 100Gbps Mellanox ConnectX-4 adapter, provided by the Kentucky Research Informatics Cloud (KyRIC). KyRIC manages the virtual machines via an OpenStack instance. We used the *targets* package to manage calculation dependencies [44]. For the comparisons of time taken using different numbers of samples to evaluate the algorithmic complexity, calculations were run on a single laptop Intel i5–10210U core clocked at 1.6 GHz.

## Results

### Datasets

In Table 1, we provide a summary of the number of features (measurements), samples, treatments or conditions, and the number of biological replicates per condition for each of the experimental datasets.

### Limit of Detection As a Cause for Missingness

We are aware of only one previous investigation of the causes of missingness in metabolomics datasets [40]. In Do et al. [40], the authors showed that there was a limit of detection (LOD) effect, with a dependence on the day the samples were run. However, one thing that seemed to be missing in the analysis was normalizing each sample for sample-to-sample variation. We feel that this is critical, given that even with measurements acquired by Metabolon (the company that performed the measurements in Do et al.), the standards do not fully correct for sample-to-sample variation. Unfortunately, the KORA4 metabolomics dataset from Do et al. is not publicly available, so we could not attempt to redo their analysis of missing values with the same dataset.

**Figure 2.**
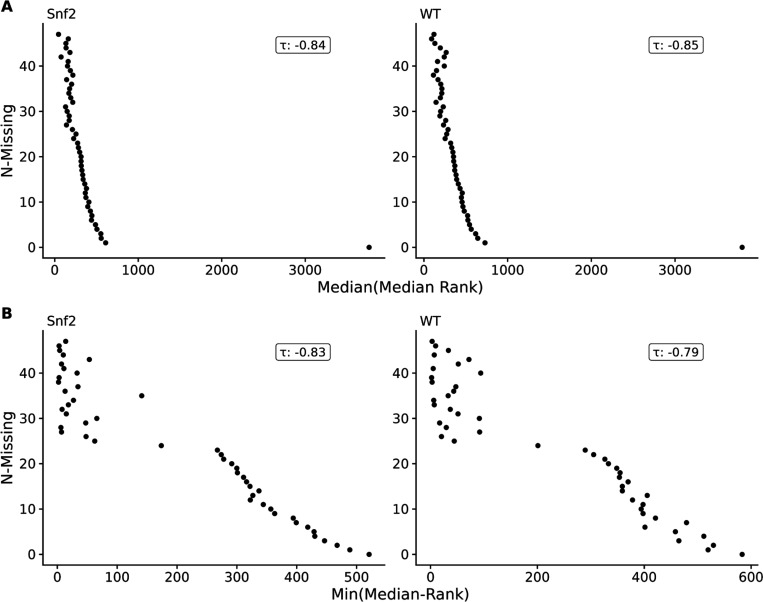
Yeast dataset of median ranks in non-missing samples by number of samples feature was missing from, using either the median (**A**) or minimum of median ranks (**B**).

An alternative to incorporating normalization, however, is to examine the median ranks of features in samples where they are present, and the number of samples they are missing from. For each experimental group of samples in each of the datasets (three RNA-seq, one metabolomics, one lipidomics), we calculated the median rank and number of measurement values missing across samples (i.e. N-Missing) for each feature (gene or metabolite, see Implementation). As shown in Figure 2A for the yeast RNA-seq dataset (also see Figures S1-S6 in Supplemental Materials for the other datasets), there is a monotonicly decreasing relationship between the median rank and the number missing values for that feature. Moreover, as N-Missing decreases, there is clearly a minimum median rank below which the values do not cross as illustrated in Figure 2B (see also Figures S1-S6 in Supplemental Materials for the other datasets). Given this relationship of the minimum median value observed and N-Present, we believe that the majority of missing values in many -omics datasets are due to being below the LOD.

This makes the ICI-Kt appropriate for use in many -omics datasets by incorporating missing values due to being below the LOD as useful information in the correlation calculation.

The LOD figures for alternative groupings of the EGFR genotype samples also demonstrate another interesting property (Figures S2-S4), in that incorrect groupings of RNA-Seq samples at the normalization stage result in negatively correlating relationships of the number of present values and the lowest observed median value as illustrated in Figure S3B.

Additional support for the hypothesis that missing values are primarily due to being below the limit of detection is provided by the binomial test for left censorship (see Implementation), with the results shown in Table 2. This test involves counting whether the non-missing values (for those features that are missing in at least one sample) are below the median of the samples where they are non-missing. For many of the datasets, the proportion of non-missing values below the median are in the range of 0.9 or higher, and only the two mass-spectrometry datasets (ratstamina and nsclc) show lower estimates. Even in those two specific cases, the proportions are 0.69 and 0.60, respectively.

### Comparison To Other Correlation Measures

We compared the ICI-Kt correlation to both Pearson and regular Kendall-tau-b correlations as calculated by the built-in R functions using simulated data sets with missing values (Figures S7-S9).

We created two samples with 1000 observations each drawn from a log-normal distribution, and sorted in each case to create a pair of X and Y samples with a correlation of 1 and −1 for both Pearson and Kendall-tau correlation measures. The *true* correlation for each of the Kendall and Pearson correlations were calculated, and then missingness was introduced in the lower range of values, up to half of the values (see Implementation).

**Figure 3.**
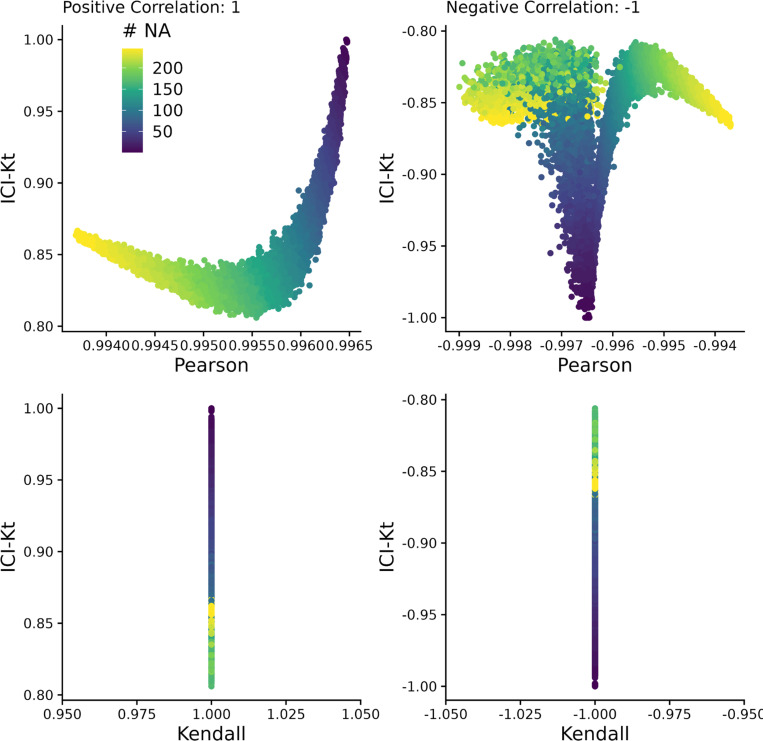
Comparing the correlation values obtained by Pearson, Kendall, and ICI-Kt correlation as an increasing number of missing values (0 – 500) in the bottom half of either sample for both positively (correlation = 1) and negatively (correlation = −1) correlated samples. Points are colored by how many points were set to missing on average between the two samples. A subset of 10,000 points was used for visualization.

In Figure 3 (and Figure S10), we can see that as missing values are added, only the ICI-Kt correlation values change in any significant way as illustrated by the wider range of ICI-Kt values on the y-axes versus the much narrower range of Pearson and Kendall tau correlation values on the x-axes. As the number of missing values increase, the ICI-Kt values drop or increase by up to 0.2. Similarly, Pearson correlation is also affected, but the degree of change in the correlation values are much less (notice the orders of magnitude differences in the x-axis scales compared to the y-axis), on the order of only 0.005 for both cases. These results demonstrate that the ICI-Kt correlation has quantitative sensitivity to missing values over the normal Kendall tau correlation and linear Pearson correlation where points with missing values are ignored (pairwise complete).

### Effect of Left Censoring VS Random Missing Data

**Figure 4.**
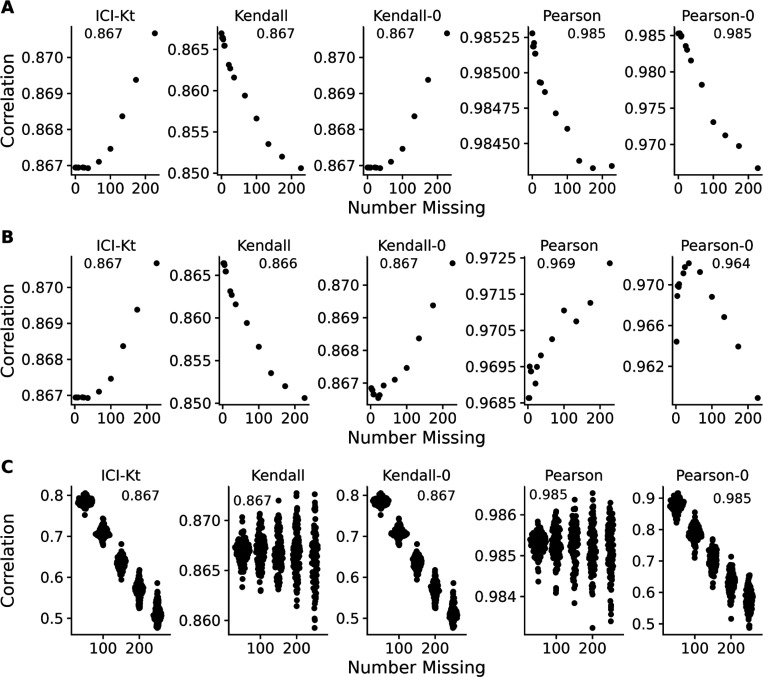
Effect of introducing missing values from a cutoff (**A** & **B**) or randomly (**C**) on different measures of correlation, including ICI-Kt, Kendall with pairwise complete, Kendall replacing missing with 0, Pearson with pairwise complete, and Pearson replacing missing with 0. **A**: Missing values introduced by setting an increasing cutoff. **B**: Missing values introduced by setting an increasing cutoff, and then log-transforming the values before calculating correlation. **C**: Missing values introduced at random. For the random case, each sample of random positions was repeated 100 times. Pay attention to the different y-axis ranges across graphs, with **A** and **B** graphs having much smaller y-axis ranges compared to **C**.

Figure 4 demonstrates the effect of introducing left-censored versus random missingness in five different measures of correlation, including the ICI-Kt, the normal Kendall-tau with *pairwise-complete-observations*, the normal Kendall-tau replacing missing with 0, Pearson with *pairwise-complete-observations*, and Pearson replacing missing with 0. The ICI-Kt correlation demonstrates a slight increase from the starting 0.86 correlation value with growing left-centered missingness caused by a slight reinforcement of the correlation, while with growing random missingness, the ICI-Kt correlation drops precipitously due to the large increase in discordant pairs caused by the random missing values. The normal Kendall tau correlation with pairwise complete has a small decrease in the correlation value with growing left-centered missingness caused by a loss of supporting pairs, while this correlation has a near constant average value with growing random missingness. The normal Kendall tau correlation replacing missing with 0 has identical behavior to the ICI-Kt correlation. In contrast to ICI-Kt, the Pearson correlation calculated using only pairwise complete entries is practically constant (i.e., range of 0.004 or less) over growing left-centered and random missingness. When replacing missing values with zero, Pearson correlation demonstrates a small decrease in the correlation value with growing left-centered missingness due to the zero values causing some deviation from linearity. Pearson correlation drops precipitously with growing random missingness with a magnitude similar to the ICI-Kt and normal Kendall tau replacing missing with 0. Overall, the ICI-Kt and the normal Kendall-tau replacing missing with zero have the desirable characteristics of maintaining the correlation with growing left-centered missing while sharply dropping the correlation with growing random missingness. In this special case where zero is lower than all of the values in the dataset, ICI-Kt and Kendall-tau replaced with zero result in identical correlation values, as shown in the bottom panels of Figure S11A and Figure S11C. In a naive treatment of the left-centered missing data, if the values below the cutoff are set to missing followed by log-transforming the values and subsequently setting missing values to 0, then the Kendall tau correlation replacing missing with 0 will show some very odd patterns at low intensity cutoffs due to the introduction of discordant pairs. Likewise, Pearson correlation replacing missing with 0 shows a parabolic effect with increasing missing values.

A common way missing data is handled in correlation calculations is to ignore them completely and use the pairwise complete cases to calculate the Pearson correlation coefficient. As shown in Figure 4C, this results in a complete misestimation of the changed correlative structure introduced by random entries. ICI-Kt, in contrast, incorporates the missingness in a sensical way, and the resulting correlation values fall as random entries are introduced.

### Differences in Dynamic Range and Correlation

Another way that missing values appear is due to changes in dynamic range between samples, as some samples have features with higher values, and the fixed dynamic range of the instrumentation results in features with lower values to be “missing” in those samples. We created a set of 100 simulated samples with uniform noise on the log-scale, with relatively constant dynamic ranges, and introduced changes to the overall dynamic range using a random censor at varying levels (see Implementation). Possible different levels of censoring based on dynamic range were checked by first determining how many missing values would be introduced in each sample as the dynamic range was increased in increments of 0.1 (see Figure S12). Based on the number of values being censored, limits of 0.5, 1, and 1.5 were selected, representing low, medium and high variability of the dynamic range.

**Figure 5.**
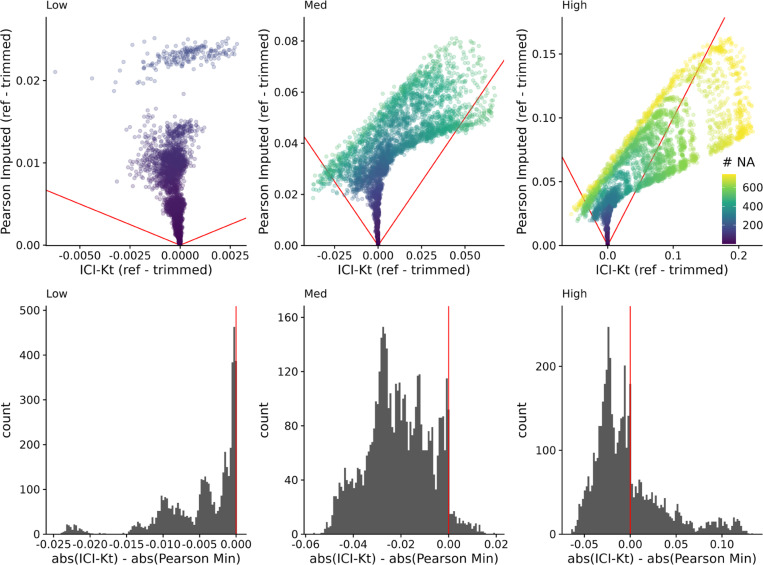
Top: Difference of reference - trimmed ICI-Kt correlation *vs* Pearson imputed using ½ the minimum value in the dataset. Low, med, and high indicate the level of variability in dynamic range, using 0.5, 1, and 1.5, respectively. Red lines indicate slope of −1 and 1. Color indicates the maximum number of missing values between the two samples being correlated. Bottom: Differences in the absolute value of reference - trimmed differences between ICI-Kt and Pearson imputed correlations.

For each level of possible missingness introduced by changes to the dynamic range, correlation across all samples were calculated using all values (reference), as well as after missingness was added (trimmed), and using Pearson correlation with global imputation (Pearson Imputed), or ICI-Kt. Figure 5 demonstrates that it is only as the number of missing values in one of the samples approaches 50% or more (500 of 1000 features) does the Pearson correlation with global imputation give correlation values closer to the known correlation with no missing values in any appreciable amount (points below the red lines in the top panels, and to the right of the red line in the histograms in the bottom panels). Points above the lines with slopes of −1 and 1 indicate that the difference of reference - trimmed is smaller in the ICI-Kt correlations, and below the lines indicate the difference is larger in the ICI-Kt correlations. This is further emphasized by the majority of the values are to the left of the line at 0 in the difference histograms.

### Performance

We compared the performance of our *Rcpp mergesort* implementation to the base R *cor* function using both “pearson” and “kendall” methods on a single core with increasing numbers of features.

**Figure 6.**
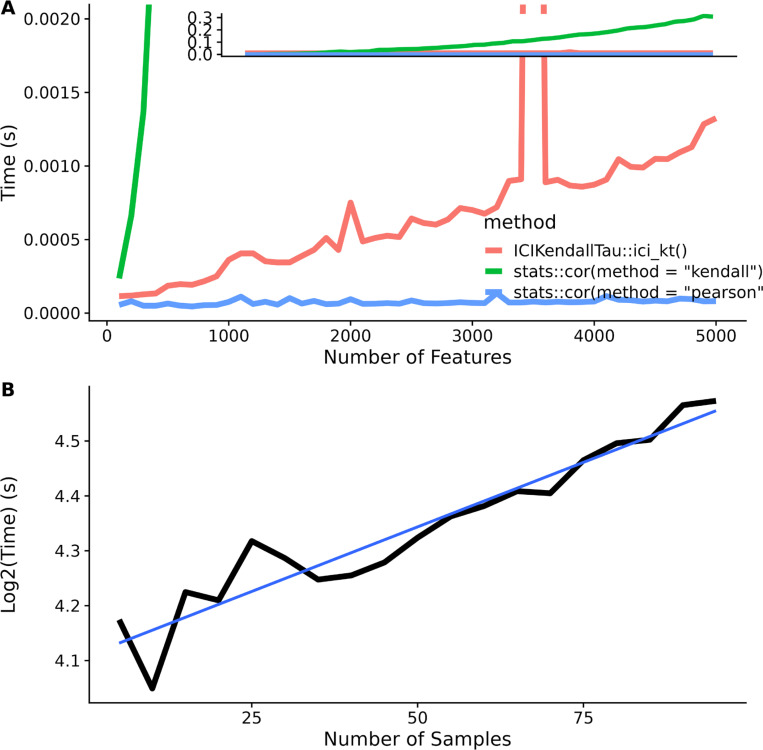
(A) Comparison of the *Rcpp mergesort ici_kt*, and base R’s *cor* function using the “pearson” and “kendall” methods as the number of features are increased from 100 to 5000. Inset is the expanded view showing the full range of computation time in seconds. (B) Log of time taken to calculate all pairwise comparisons using ICI-Kt for different numbers of samples in multicore processing.

Figure 6A shows that base R cor “kendall” method is the slowest by far. Zoomed out (inset), the ICI-Kt appears as fast as using “pearson”, but upon zooming in, it’s possible to see that ICI-Kt is using increasing amounts of time much faster than “pearson”. In fact, regressing the time to number of samples using a formula with the expected algorithmic complexity, “pearson” shows O(n), ICI-Kt is O(nlog(n)), and “kendall” is O(n^2) (see Figure S13).

As we implemented a relatively easy way to make use of asynchronous compute resources via the *future* package, we also verified that increases in run time using multiple cores was reasonable as well. Figure 6B shows that as samples are added, the run time quickly increases logarithmically, similar to increasing samples on a single core (see Figure 6A).

### Utility for Large Omics’ Data Sets

#### Detecting Outlier Samples

We analyzed datasets from RNA-Seq (3), lipidomics (1), and small molecule metabolomics (1) for possible outliers using a variety of different correlation and correlation-related metrics (see Correlation Methods): (1) ICI-Kt (IK); (2) ICI-Kt × completeness (ie., ICI-Kt scaled by completeness; see ICK in Correlation Methods); (3) Kendall-tau (Kt); (4) Pearson correlation (PB); (5) Pearson correlation with no zeros (PN0); (6) Pearson on log(x+1) (PL1); and (7) Pearson on log(x) (PL). We primarily concentrate on the ICI-Kt, IKC, and Pearson log(x+1) here for one dataset, but all outliers detected by each method are shown in the Supplemental materials (Figures S14-S18, Tables S1-S6). For each dataset and correlation-related metric, the median correlation for a sample was calculated amongst the samples for each sub-group of samples corresponding to an experimental condition of interest (disease, mutant, condition, etc).

**Figure 7.**
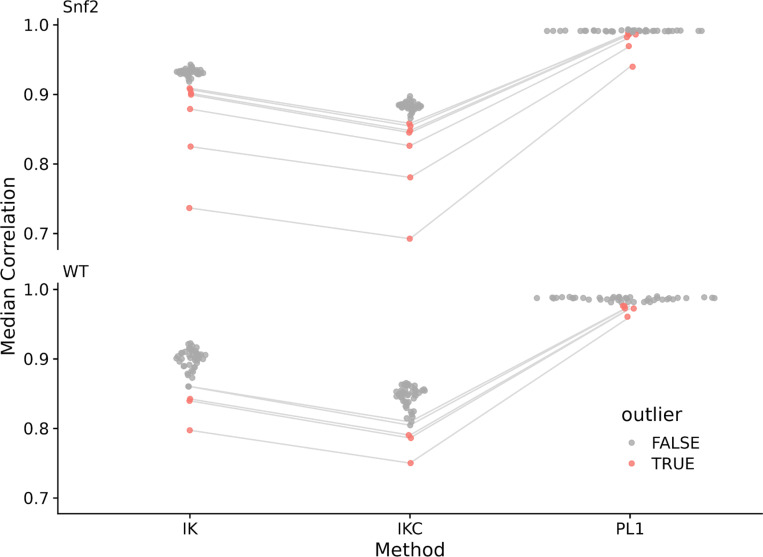
Median correlations for each of the yeast RNA-Seq sample to all other samples in the same group, using either ICI-Kt (IK), ICI-Kt × Completeness (IKC) or Pearson Log(x+1) (PL1) correlation. Points are colored red if they were an outlier using that correlation method. For a sample that is considered an outlier in any of the methods, lines are drawn connecting them between methods.

Figure 7 visualizes the sample-specific median of each correlation-related metric calculated from the Yeast RNA-Seq data set. Table 3 lists the outliers detected based on each sample-specific median metric value.

In this example, ICI-Kt shows superior sensitivity for outlier detection as compared to the other metrics and is likely related to the increased spread of median metric values as compared to Pearson. We also note that these outliers seem to be a superset of the outliers noted by Gierliński et al. (see Figure S14 and Table S1).

Gierliński et al [14] proposed a combination of median sample-sample correlation, gene count outlier fraction, and a chi-square test of read coverage per gene to score each biological replicate from a group of samples. Outside of defining this combined score, they do not describe the actual criteria of saying a replicate is “bad”. In this work, we used only the sample-sample median correlation to identify “bad” or “outlier” samples, using the *boxplot.stats* function to determine outliers, where an outlier is defined as samples that are greater than 1.5X away from the limits of the box defining the distribution.

Here, we calculated Pearson correlation using the raw counts, and with only those genes that had non-zero reads in both samples (also see PN0). This version of Pearson correlation recreates the median sample-sample correlations observed in the original report. We denote the actual samples recorded as outliers by Gierliński and coworkers with “Manuscript”. In Figure S14 and Table S1, we can see how the determination of outliers was not made solely on the basis of correlation alone, but on a combination of factors that lead to some of the higher correlating samples (using raw counts and Pearson correlation) being considered outliers where lower correlating samples were not listed as being outliers.

Regardless, using ICI-Kt or ICI-Kt × Completeness in this instance, the outliers using the simple distribution summary statistics and an outlier having to be greater than 1.5X median error, the outliers are mostly a superset of the outliers determined by Gierliński et al., with the exception of three samples specific to their data: WT.22, WT.25, WT.28.

It should be noted that it seems that Gierliński et al. used an eyeball cutoff for the outliers based on the combined score of correlation, outlier fraction, and read coverage chi-square fitness, with samples having a combined log-score greater than −2.8 (evaluated by RMF eyeball on a zoomed in graph and using a ruler), a value that is never stated in the manuscript, and which seems arbitrary from the data. In addition, given the proportional variance inherent in RNA-Seq count data, Pearson correlation on non-transformed counts should not be used, as the low abundance counts will tend to force the correlation values to be arbitrarily high in the absence of large biological heterogeneity, as observed for these samples compared to the other datasets that involve individuals as biological replicates.

In the case of the adenocarcinoma dataset (Figure S16 and Table S3), although there are not many outliers in these relatively large groups, Kendall-tau (Kt) values results in the most outliers, mostly in the *tumor* samples. Also of note is that ICI-Kt x Completeness (IKC) is the only measure where the variance measure for both groups is similar (Table S4) (1.8X). For both ICI-Kt and the various Pearson correlation, the tumor samples variance measures are 3.1X and 3.6X greater than normal, respectively.

Additionally, we compared how the outliers change if we require that a feature is present in at least a certain fraction of samples of a class with each different correlation measure (see Figures S19-S33). Although, in general the correlations remain the same, or show some downward trend with increasing fractions of samples required to be non-missing, IKC shows a different trend. Overall, this is due to there being a higher likelihood of pairwise samples being *complete* with the requirement for a feature to be in a greater fraction of a class of samples.

With the exception of the yeast dataset, the base Pearson (PB) and Pearson without zeros (PN0) were the most likely methods to show higher changes in median and median-absolute-differences (MAD) between groups of samples, as shown in Figures S21, S24, S27, S30 and S33 (see Implementation).

### Partitioning of Feature-Feature Networks

Feature-feature correlations (for those features present in 25% or more of the samples of a class) for each dataset were calculated using each of the correlation methods (see Implementation). From those feature-feature correlations, partial correlations are calculated, and significant edges retained. Feature annotations from either Reactome pathways (RNA-Seq), KEGG pathways (metabolomics), or lipid class (lipidomics) were used to determine if the edges within annotations were more present than outside of annotations. The biochemical rationale for this analysis is that stronger positive partial correlations should be reflected within known pathways versus between pathways.

Table 4 lists the summed partitioning values for each dataset and method. For each of the datasets examined, ICI-Kt or ICI-Kt completeness gave the most positive summed partitioning values.

## Discussion and Conclusion

Left-censored distributions in analytical measurements of biological samples are common in biological and biomedical research, because of detection limits of the analytical instrumentation, which produces missing measurement values for all the analytes below these detection limits. As far as we are aware, previous efforts in this area are concerned with either 1: attempting to come up with better imputation methods prior to calculating correlation values; or 2: finding ways to still estimate the correlation in the face of missing values, generally by finding maximum likelihood estimates. In the case of (1), there are many imputation methods, and new methods are still being developed, although they tend to be new combinations of old methods to handle the various types of missing values. For (2), the maximum likelihood methods generally apply to Pearson and similar types of correlation, as they benefit from the use of regression in the presence of missing data. Alvo and Cabilio’s work from 1995 [45] is one of the only works we are aware of that attempts to create a general framework for rank-based statistics in the presence of missing data. But, in our understanding their framework applies to data that is missing at random versus non-random missing-values, as is the case for analytes that are below the detection limit. Additionally, there does not appear to be a software implementation of Alvo and Cabilio’s method available.

Although the actual implementation of the base ICI-Kt correlation metric involves a global imputation of missing values, our equations demonstrate a left-censorship interpretation of missing values as useful information within the calculated correlation. Furthermore, the addition of “local”, “global” and $tau_{max}$ normalizations of the ICI-Kt correlation in combination with Completeness provide additional interpretations of information content. Finally, the use of the binomial left-censorship test ensures the application of the ICI-Kt methodology when it is appropriate.

Global imputation methods rely on the assumption that samples have similar dynamic range of detection and thus an imputed value should be comparable between samples. However, the dynamic range of detection often has variability across samples. For complex analytical techniques often used in omics experiments, the variability in the dynamic range of detection can be quite high. Under these circumstances, the ICI-Kt method provides more robust results as compared to Pearson correlation with global imputation. This holds true for low, medium, and high variability in dynamic range across samples.

In the case of using sample-sample correlation to detect outliers, imputation does not solve any of the issues related to discovering outliers, as it should be applied after outlier samples are removed, otherwise the imputed values may not be useful. When used to create feature-feature networks based on partial correlations derived from the feature-feature correlations, ICI-Kt based methods also showed the best partitioning of features based on their known pathway annotations. As far as we know, information-content-informed Kendall-tau (ICI-Kt) is the first correlation method that explicitly attempts to utilize non-random missing values that occur due to being below the detection limit. ICI-Kt explicitly treats left-censored missing values as correlative information while preserving the full deleterious effects of missing at random values on the correlation metric. Moreover, ICI-Kt can be combined with measurement completeness to create a composite metric that is quite sensitive to overall data quality on a sample-specific level. Also, this ICI-Kt × completeness metric may have applications in cluster detection of single-cell omics data sets.

The implementations of the ICI-Kt method in the presented R and Python packages provide a rich set of options for customizing the correlation calculation for a variety of use-cases and interpretations. These packages handle missing values in log-transformed data in a safe manner and have O(nlogn) performance, making them computationally practical for real-world omics data sets. Also, these packages provide multiprocessing implementations that take full advantage of modern multi-core central processing units.

As demonstrated with the datasets analyzed here, the “best” correlation-related metric will likely depend on the specific dataset and the specific data analysis step. Many factors affect this, especially correlation linearity and the modality of measurement value distributions. We would humbly suggest that for most omics datasets, the application of several correlation-related metrics simultaneously would be the best approach for outlier detection in quality control and quality assessment steps. Where one metric lacks outlier detection sensitivity, another metric will prove sensitive. Therefore, ICI-Kt and associated composite metrics should be considered as useful additions to the omics data analysis toolkit.

## Supplementary Material

Supplement 1

In addition to the over 30 supplemental figures and tables, the following Zenodo item includes all of the data and code used to generate results: https://zenodo.org/doi/10.5281/zenodo.6309187

## Figures and Tables

**Table 1.** Number of features, samples, treatments, and biological replicates for each dataset.

Dataset	Measurement	Features	Samples	Treatments	Replicates
yeast	RNA-Seq	6,887	96	2	48, 48
adenocarcinoma	RNA-Seq	57,655	264	2	28, 236
egfrgenotype	RNA-Seq	30,875	56	3	18, 20, 18
egfrgenotypetumorculture	RNA-Seq	30,875	56	4	20, 14, 11, 11
typeandtumorculture	RNA-Seq	30,875	56	12	6, 4, 5, 3, 7, 5, 3, 5, 7, 5, 3, 3
ratstamina	Metabolomics	532	42	4	9, 12, 12, 9
nsclc	Lipidomics	29,672	146	4	38, 39, 35, 34

**Table 2.** For each dataset, the total number of elements, number of elements with a missing value, % missing values, number of values tested as being below the median in a sample, number of values found to be below the median of a sample, fraction of values found to be below the median of sample, confidence interval, and p-value of the left-censored binomial test. In each case, the reported p-value is below 2.2 × 10^−16^, and represented as 0 in the table.

Dataset	Total	Missing	% Missing	Trials	Success	Estimate	CI	P-Value
yeast	6.61×10^5^	2.79×10^4^	4%	3.88×10^4^	3.88×10^4^	1.00	1.00 – 1.00	0
adeno carcinoma	1.52×10^7^	4.85×10^6^	32%	4.24×10^6^	4.01×10^6^	0.95	0.94 – 1.00	0
egfrgenotype	1.73×10^6^	8.82×10^5^	51%	2.08×10^5^	1.87×10^5^	0.90	0.90 – 1.00	0
egfrgenotype tumorculture	1.73×10^6^	8.82×10^5^	51%	7.89×10^4^	7.79×10^4^	0.99	0.99 – 1.00	0
typeandtumor culture	1.73×10^6^	8.82×10^5^	51%	5.07×10^4^	5.03×10^4^	0.99	0.99 – 1.00	0
ratstamina	2.23×10^4^	9.70×10^3^	43%	6.63×10^3^	4.56×10^3^	0.69	0.68 – 1.00	0
nsclc	4.33×10^6^	3.94×10^6^	91%	3.21×10^5^	1.91×10^5^	0.60	0.60 – 1.00	0

**Table 3.** Yeast dataset median correlation values and outlier determination for each outlier from each of ICI-Kt (IK), ICI-Kt × completeness (IKC) or Pearson Log(x+1) (PL1) correlation methods. Bolded entries indicate the sample was an outlier for that method.

Sample	IK	IKC	PL1
Snf2.10	**0.907**	**0.855**	**0.989**
Snf2.31	**0.902**	**0.848**	**0.987**
Snf2.35	**0.909**	**0.858**	**0.986**
Snf2.15	**0.900**	**0.845**	**0.986**
Snf2.25	**0.879**	**0.826**	**0.982**
Snf2.13	**0.825**	**0.781**	**0.970**
Snf2.06	**0.737**	**0.693**	**0.940**
WT.36	0.860	0.810	**0.976**
WT.28	0.860	0.805	**0.976**
WT.25	**0.843**	**0.790**	**0.973**
WT.34	**0.840**	**0.786**	**0.973**
WT.21	**0.797**	**0.750**	**0.961**

**Table 4.** Partitioning ratios for feature-feature correlation networks generated using each method for each dataset. Bolded entries indicate the highest partitioning ratio for the dataset.

Method	Yeast RNA-Seq	EGFR Genotype RNA-Seq	Rat Stamina Metabolomics	NSCLC Lipidomics
IK	**−0.918**	**−0.720**	0.045	−0.176
IKC	−0.935	−1.011	**0.395**	**−0.133**
Kt	−0.943	−0.785	−0.756	−0.295
PB	−1.001	−0.844	0.009	−0.155
PL1	−1.008	−0.858	−0.055	−0.186
PN0	−3.317	−0.885	−0.703	−0.319
PL		−0.952	−0.373	−0.288

## Data Availability

GitHub repository for the R package: https://github.com/moseleyBioinformaticsLab/ICIKendallTau GitHub repository for the Python package: https://github.com/moseleyBioinformaticsLab/icikt Python Package Index: https://pypi.org/project/icikt/ Zenodo Item: https://zenodo.org/doi/10.5281/zenodo.6309187
