# Supplementary material for: Information-Content-Informed Kendall-tau Correlation Methodology: Interpreting Missing Values as Useful Information
Source: bioRxiv. 2025 Jul 21:2022.02.24.481854. Preprint. [Version 4] doi: 10.1101/2022.02.24.481854 (PMC12330630; doi:10.1101/2022.02.24.481854)
Supplement: Supplement 1 [file NIHPP2022.02.24.481854v4-supplement-1.pdf]

bioRxiv preprint doi: https://doi.org/10.1101/2022.02.24.481854; this version posted July 21, 2025. The copyright holder for this preprint (which was not certified by peer review) is the author/funder, who has granted bioRxiv a license to display the preprint in perpetuity. It is made available under aCC-BY 4.0 International license.

Supplemental Materials

Robert M Flight, Praneeth S Bhatt, and Hunter NB Moseley

2025-07-21 13:18:17.884816

Limits of Detection Issues in Datasets

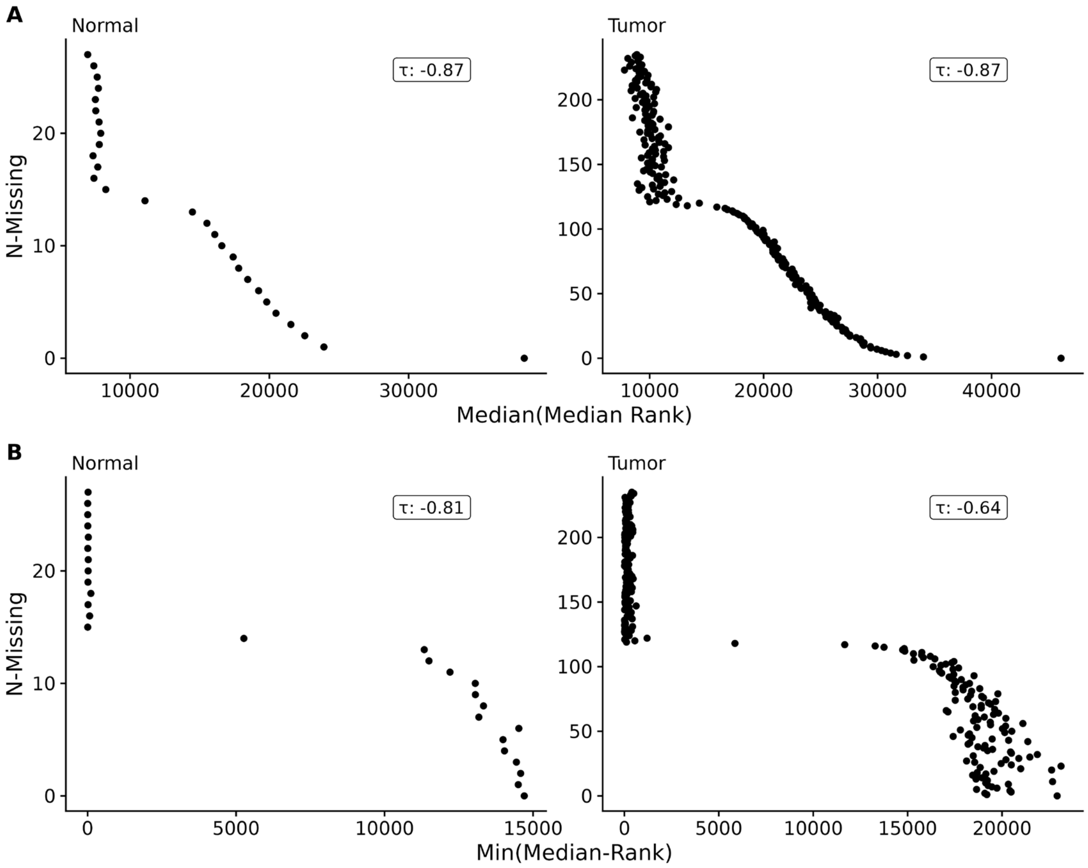

Figure S1. Adenocarcinoma dataset of median ranks in non-missing samples by number of samples feature was missing from, using either the median (**A**) or minimum of median ranks (**B**).

bioRxiv preprint doi: https://doi.org/10.1101/2022.02.24.481854; this version posted July 21, 2025. The copyright holder for this preprint (which was not certified by peer review) is the author/funder, who has granted bioRxiv a license to display the preprint in perpetuity. It is made available under aCC-BY 4.0 International license.

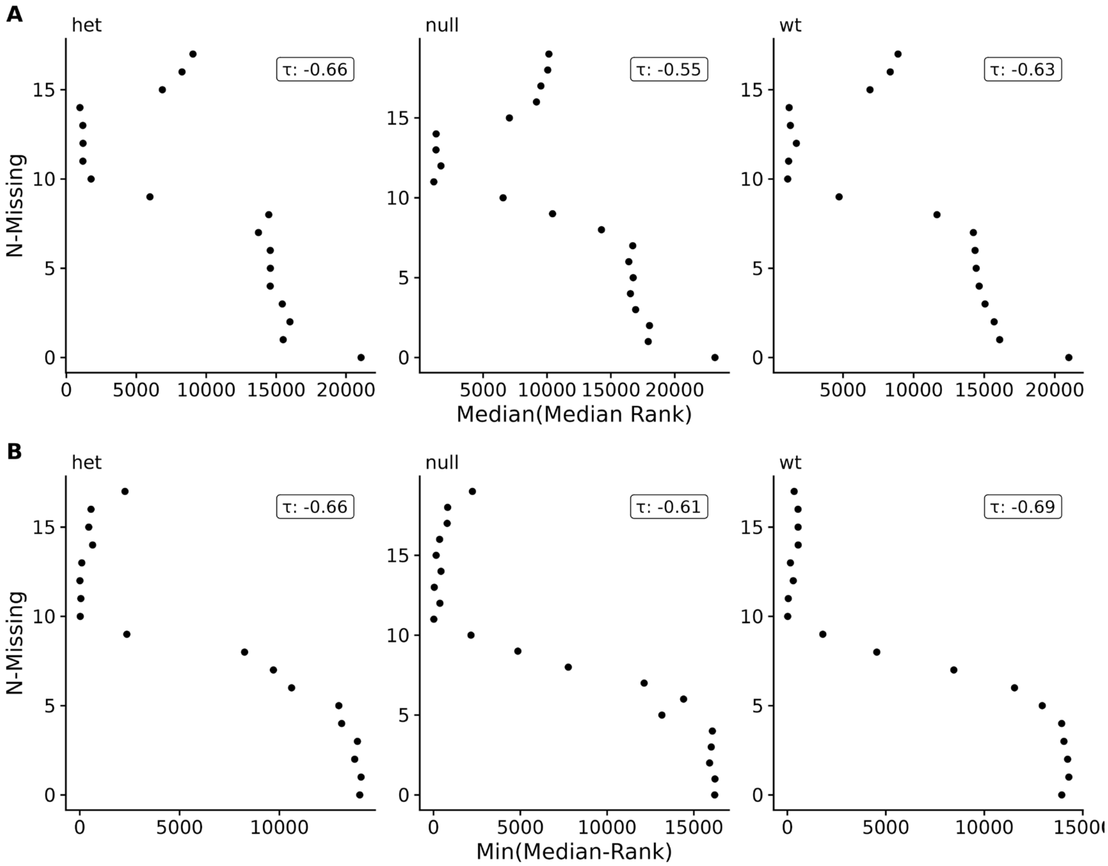

Figure S2. EGFR genotype dataset of median ranks in non-missing samples by number of samples feature was missing from, using either the median (**A**) or minimum of median ranks (**B**).

bioRxiv preprint doi: https://doi.org/10.1101/2022.02.24.481854; this version posted July 21, 2025. The copyright holder for this preprint (which was not certified by peer review) is the author/funder, who has granted bioRxiv a license to display the preprint in perpetuity. It is made available under aCC-BY 4.0 International license.

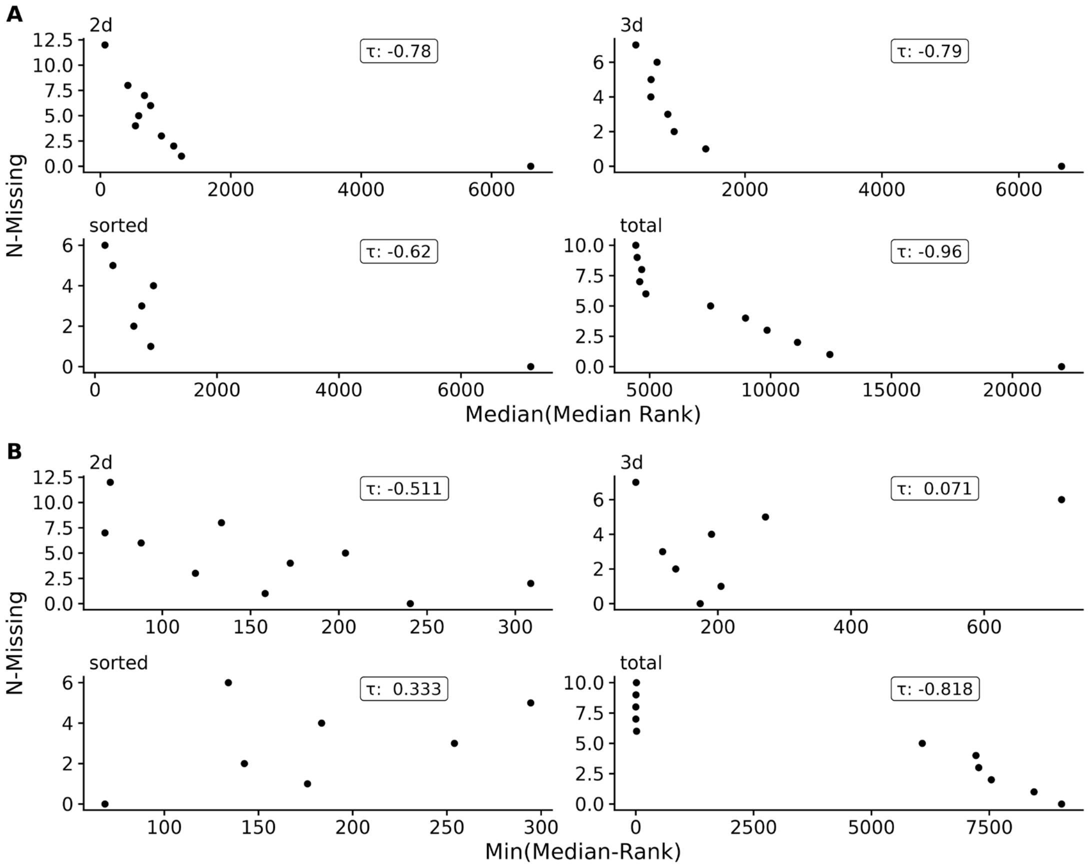

Figure S3. EGFR genotype and tumor culture dataset dataset of median ranks in non-missing samples by number of samples feature was missing from, using either the median (**A**) or minimum of median ranks (**B**).

bioRxiv preprint doi: https://doi.org/10.1101/2022.02.24.481854; this version posted July 21, 2025. The copyright holder for this preprint (which was not certified by peer review) is the author/funder, who has granted bioRxiv a license to display the preprint in perpetuity. It is made available under aCC-BY 4.0 International license.

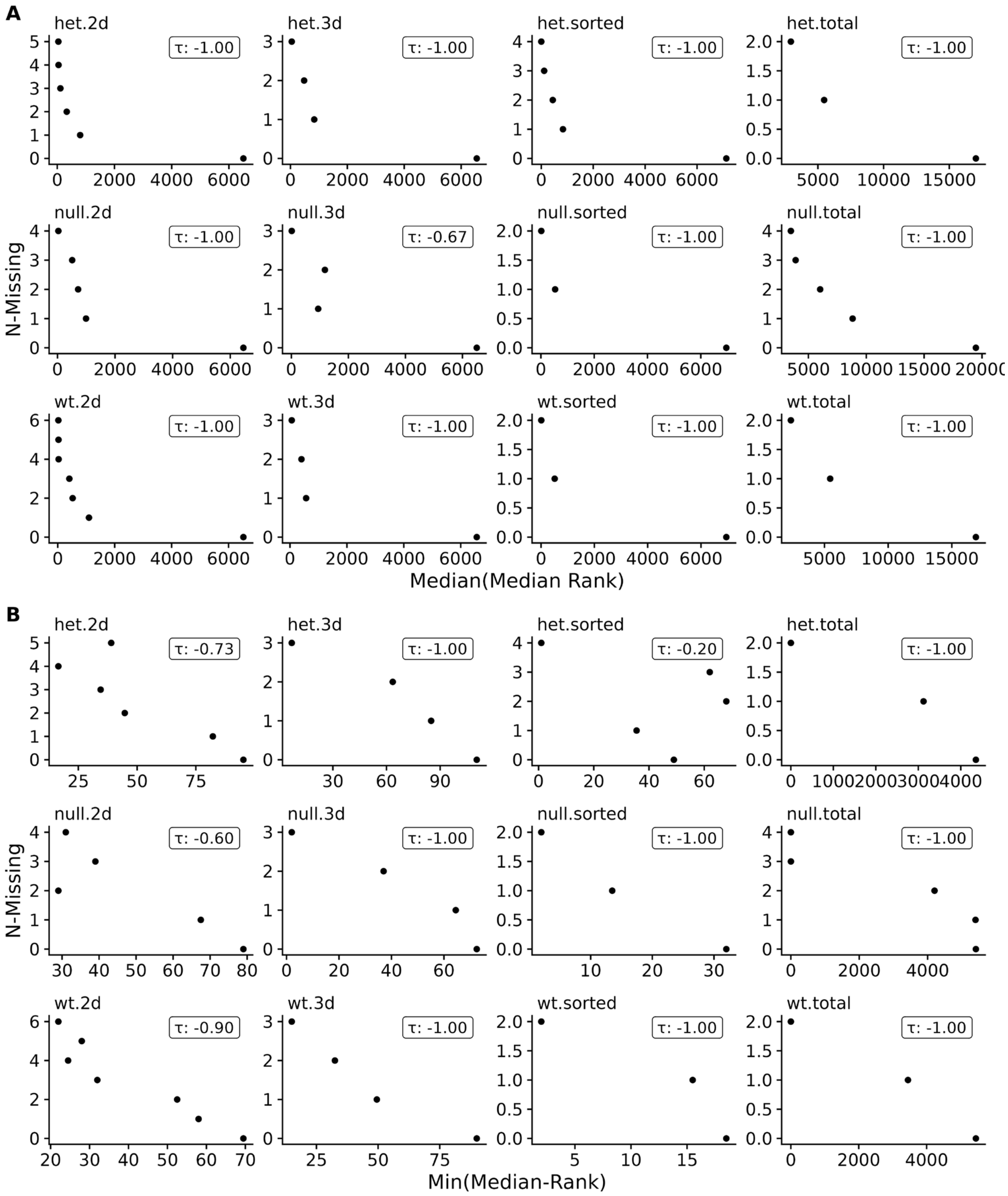

Figure S4. EGFR genotype and tumor culture dataset of median ranks in non-missing samples by number of samples feature was missing from, using either the median (**A**) or minimum of median ranks (**B**).

bioRxiv preprint doi: https://doi.org/10.1101/2022.02.24.481854; this version posted July 21, 2025. The copyright holder for this preprint (which was not certified by peer review) is the author/funder, who has granted bioRxiv a license to display the preprint in perpetuity. It is made available under aCC-BY 4.0 International license.

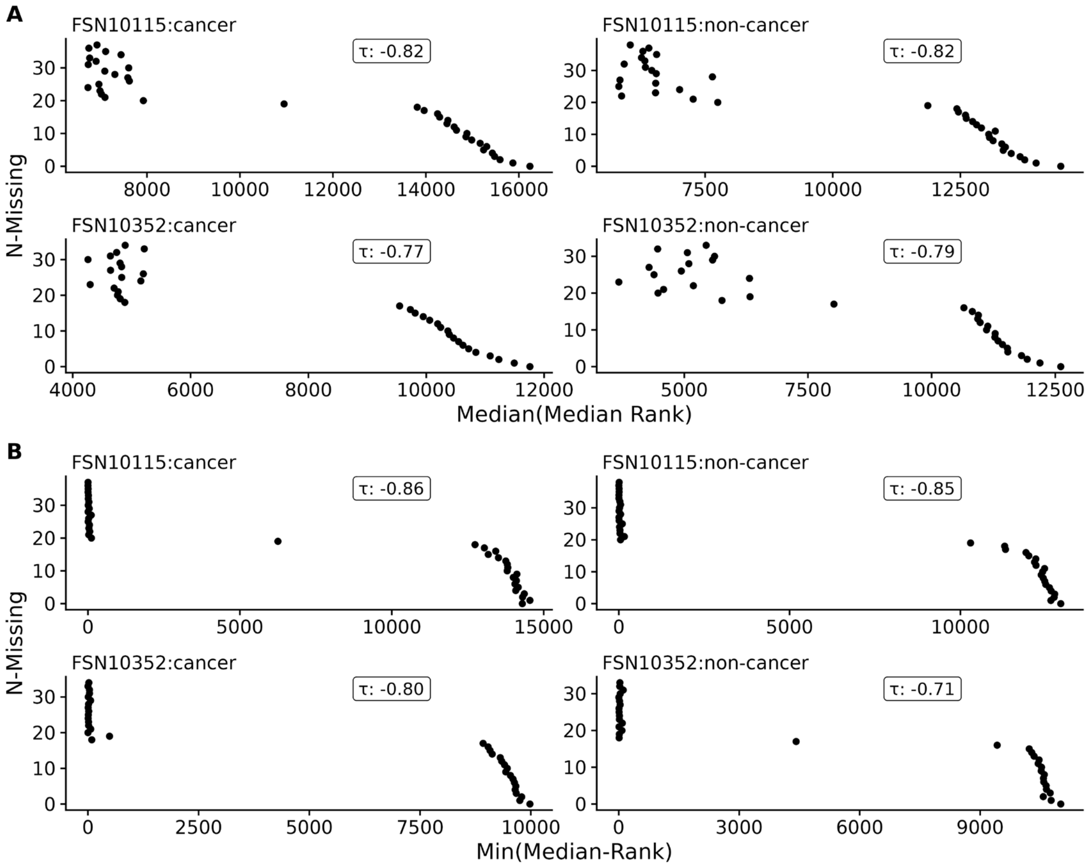

Figure S5. Non-small-cell-lung-cancer (NSCLC) lipidomics dataset of median ranks in non-missing samples by number of samples feature was missing from, using either the median (**A**) or minimum of median ranks (**B**).

bioRxiv preprint doi: https://doi.org/10.1101/2022.02.24.481854; this version posted July 21, 2025. The copyright holder for this preprint (which was not certified by peer review) is the author/funder, who has granted bioRxiv a license to display the preprint in perpetuity. It is made available under aCC-BY 4.0 International license.

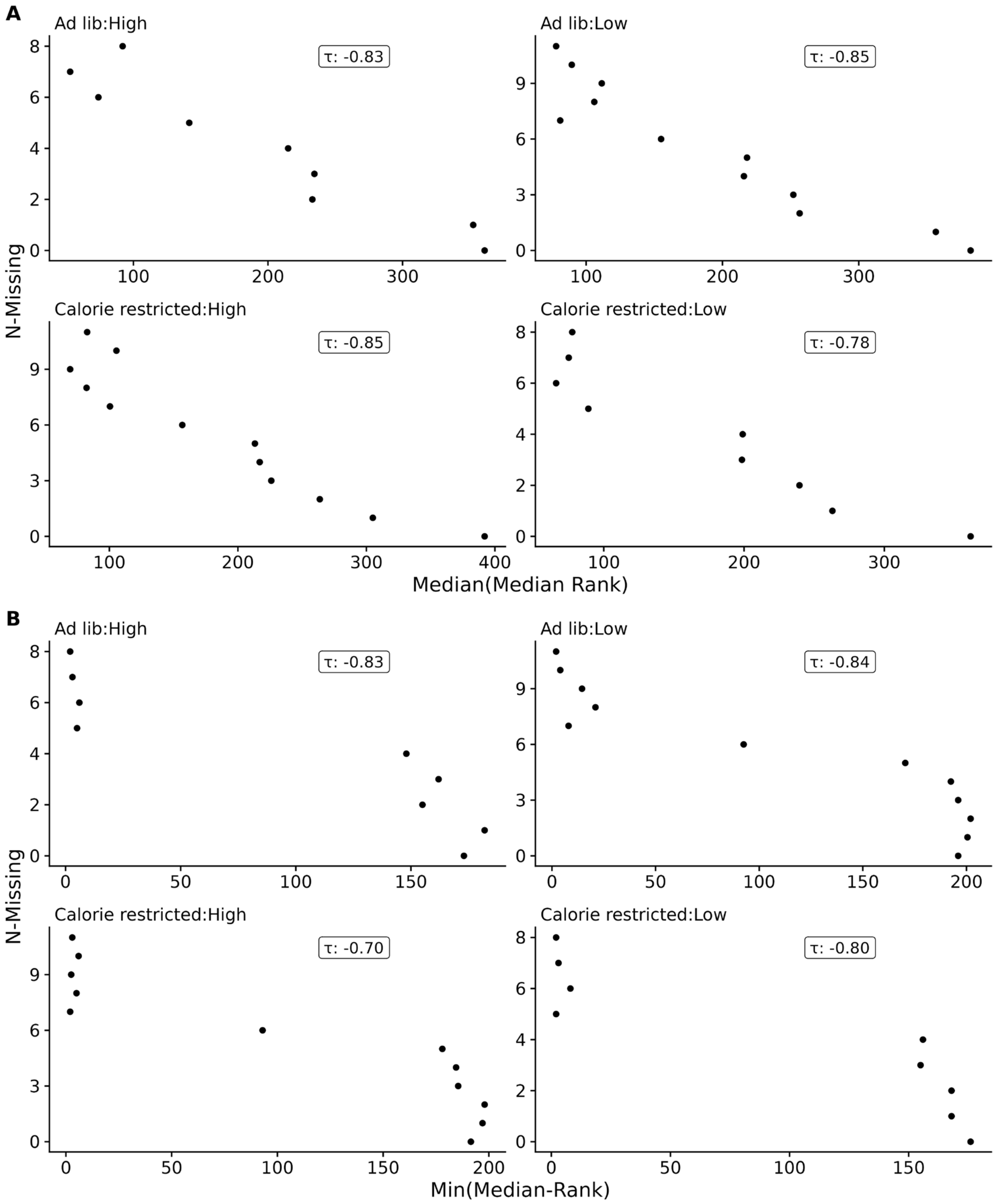

Figure S6. Rat stamina dataset of median ranks in non-missing samples by number of samples feature was missing from, using either the median (**A**) or minimum of median ranks (**B**).

bioRxiv preprint doi: https://doi.org/10.1101/2022.02.24.481854; this version posted July 21, 2025. The copyright holder for this preprint (which was not certified by peer review) is the author/funder, who has granted bioRxiv a license to display the preprint in perpetuity. It is made available under aCC-BY 4.0 International license.

Simulated Data

Simple Data Set

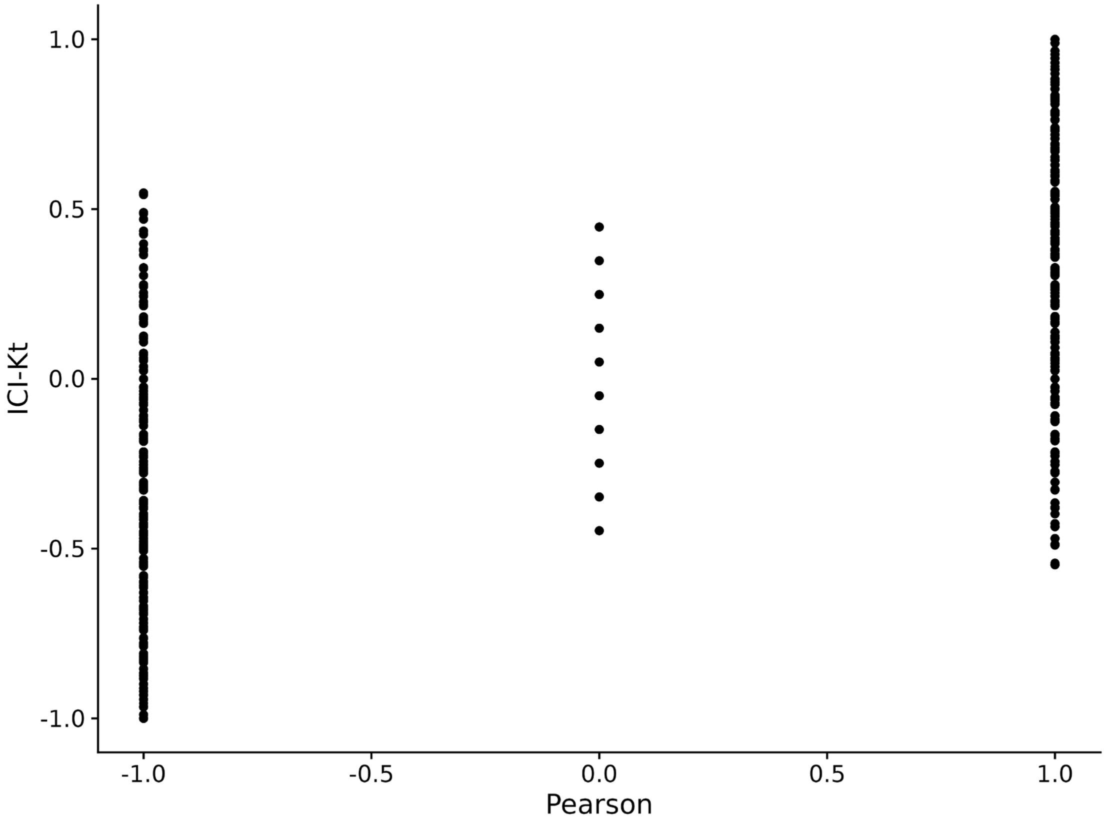

Figure S7. Comparison of ICI-Kt and Pearson correlations for perfectly positive and negatively correlated samples, systematically replacing values with NA. NA values from Pearson were replaced with zero for this comparison.

We can also examine the full set of positive and negative correlations generated as we vary the number of missing entries between two positively correlated samples and two negatively correlated samples. These distributions are shown in Figure S9. We can see that the distributions from both ICI-Kt and Kendall-tau are the same, which is expected given we replaced missing values (NA) with zero *within* the ICI-Kt code, and replaced missing values (NA) with zero prior to calculating Kendall-tau correlations.

bioRxiv preprint doi: https://doi.org/10.1101/2022.02.24.481854; this version posted July 21, 2025. The copyright holder for this preprint (which was not certified by peer review) is the author/funder, who has granted bioRxiv a license to display the preprint in perpetuity. It is made available under aCC-BY 4.0 International license.

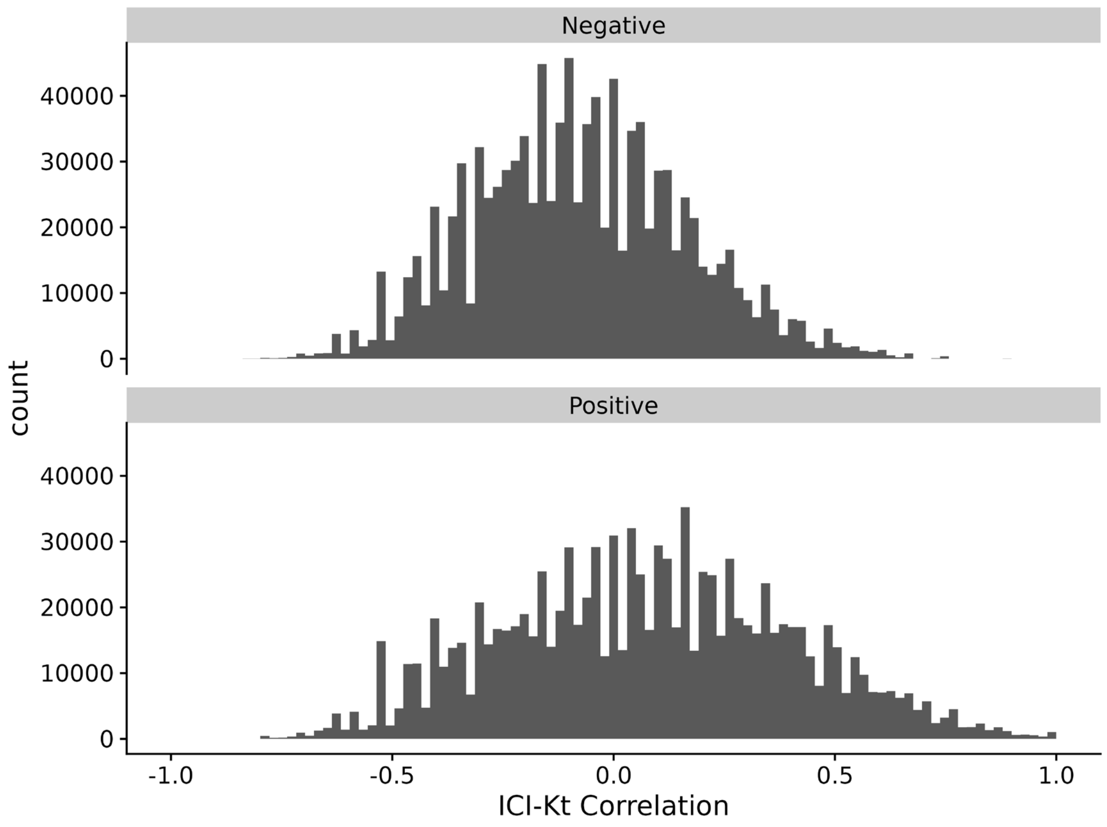

Figure S8. ICI-Kendall-tau correlation as missing values are varied between two samples.

bioRxiv preprint doi: https://doi.org/10.1101/2022.02.24.481854; this version posted July 21, 2025. The copyright holder for this preprint (which was not certified by peer review) is the author/funder, who has granted bioRxiv a license to display the preprint in perpetuity. It is made available under aCC-BY 4.0 International license.

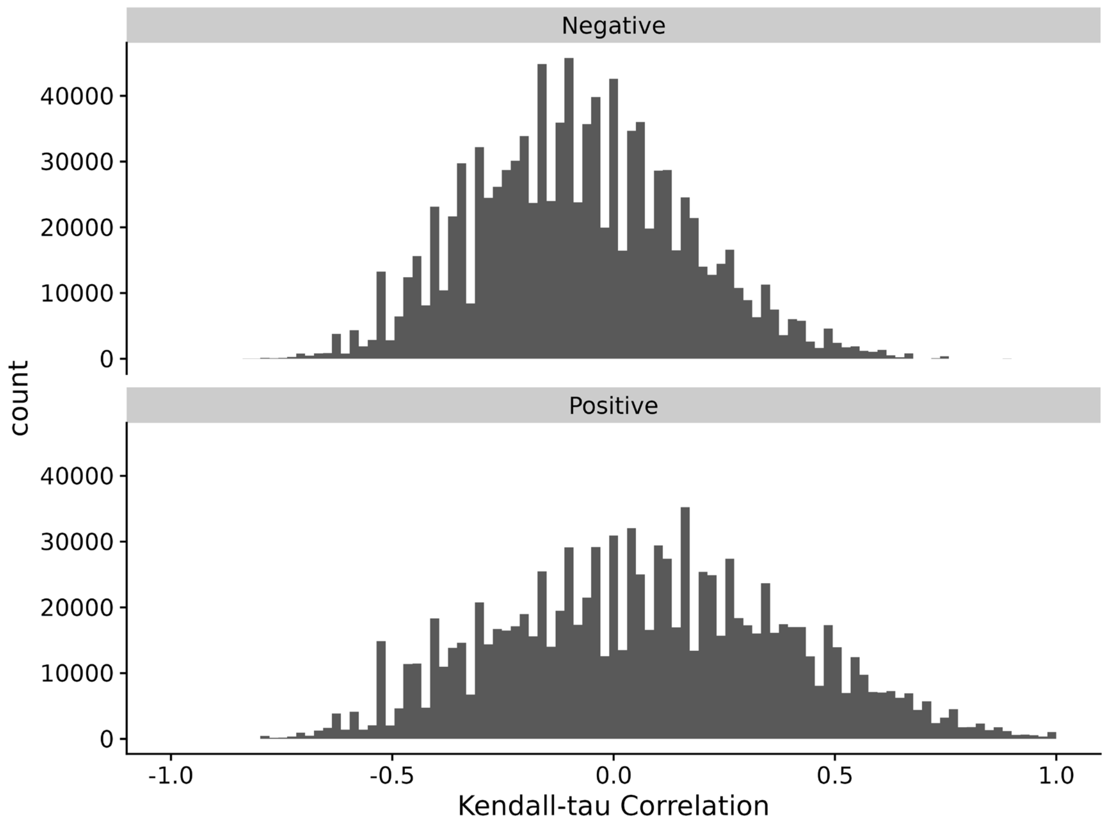

Figure S9. Kendall-tau correlation as missing values are varied between two samples and replaced with 0 before calculating Kendall-tau.

bioRxiv preprint doi: https://doi.org/10.1101/2022.02.24.481854; this version posted July 21, 2025. The copyright holder for this preprint (which was not certified by peer review) is the author/funder, who has granted bioRxiv a license to display the preprint in perpetuity. It is made available under aCC-BY 4.0 International license.

Comparison to Other Correlation Measures

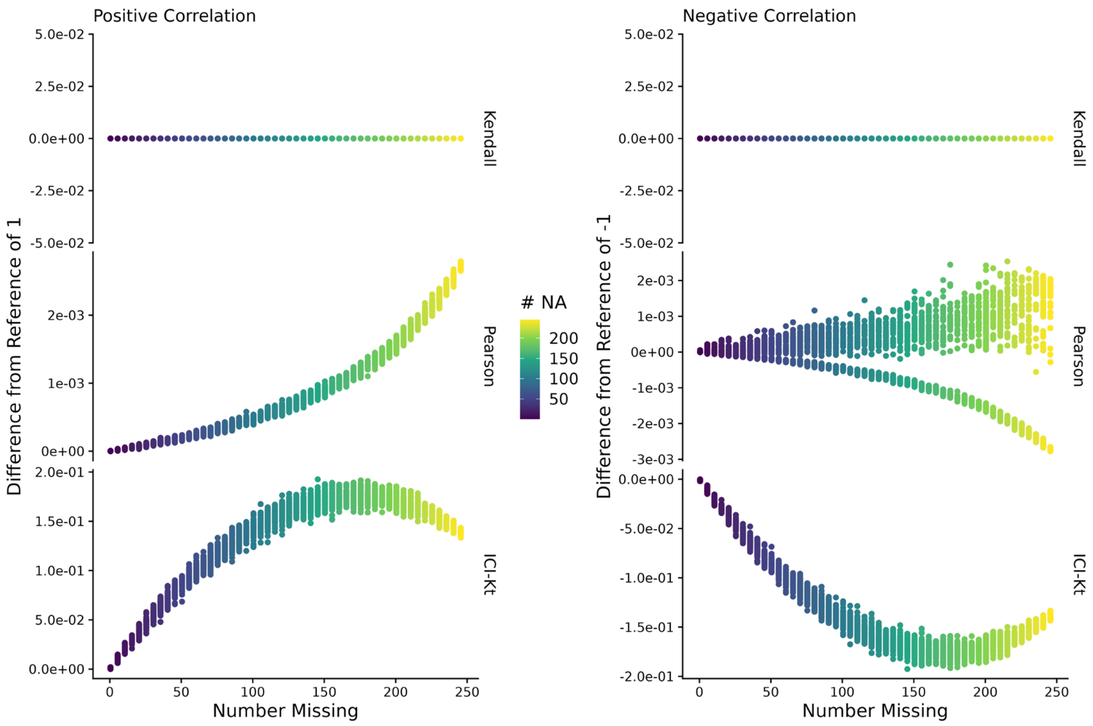

Figure S10. Difference of estimated correlation with missingness introduced compared to a reference correlation of 1 for the positive or -1 for the negative case, as a function of the average number of missing entries in X and Y sample (# NA). Points are colored by how many points are missing on average between the two samples X and Y.

bioRxiv preprint doi: https://doi.org/10.1101/2022.02.24.481854; this version posted July 21, 2025. The copyright holder for this preprint (which was not certified by peer review) is the author/funder, who has granted bioRxiv a license to display the preprint in perpetuity. It is made available under aCC-BY 4.0 International license.

Semi-Realistic Data Set

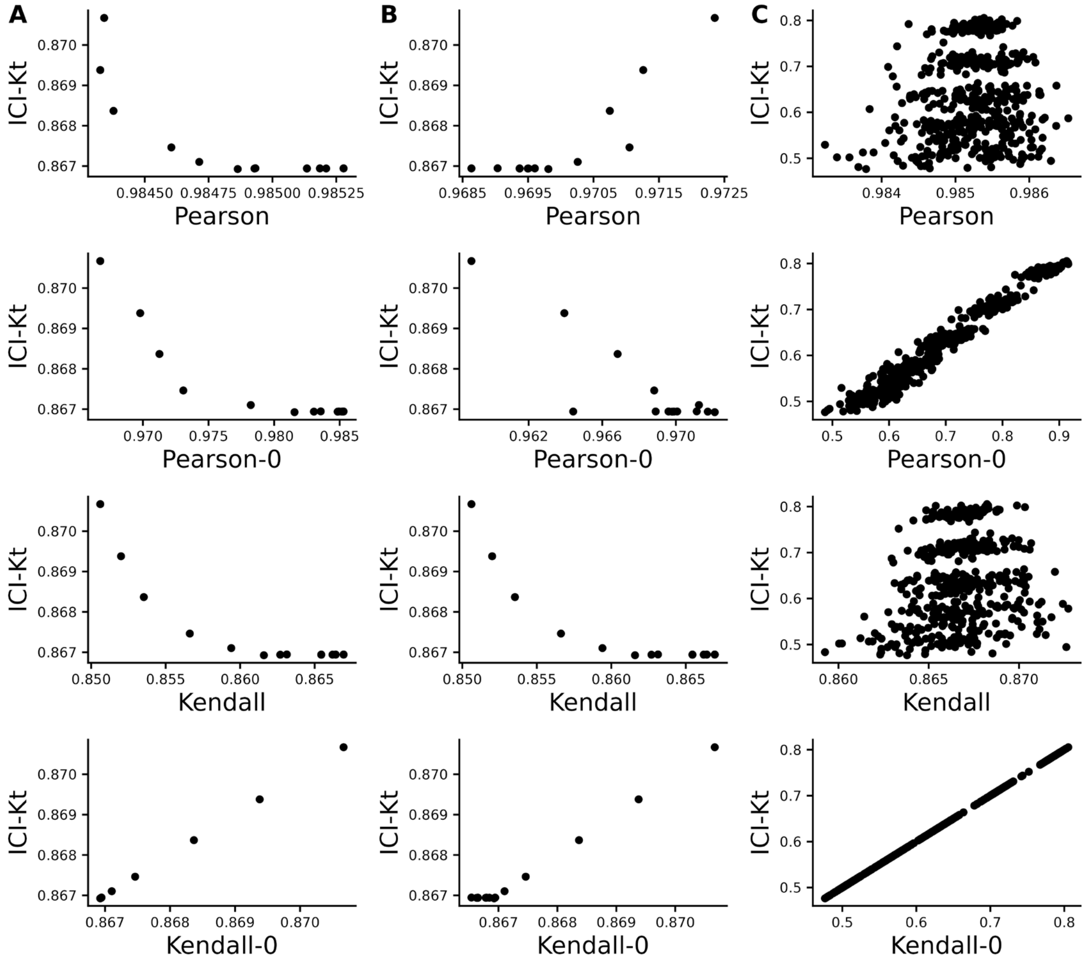

Figure S11. Effect of introducing missing values from a cutoff (A & B) or randomly (C) on different measures of correlation, including ICI-Kt, Kendall with pairwise complete, Kendall replacing missing with 0, Pearson with pairwise complete, and Pearson replacing missing with 0. A) Missing values introduced by setting an increasing cutoff. B) Missing values introduced by setting an increasing cutoff, and then log-transforming the values before calculating correlation. C) Missing values introduced at random. For the random case, each sample of random positions was repeated 100 times.

bioRxiv preprint doi: https://doi.org/10.1101/2022.02.24.481854; this version posted July 21, 2025. The copyright holder for this preprint (which was not certified by peer review) is the author/funder, who has granted bioRxiv a license to display the preprint in perpetuity. It is made available under aCC-BY 4.0 International license.

Changes In Correlation Due to Changes in Dynamic Range and Imputation

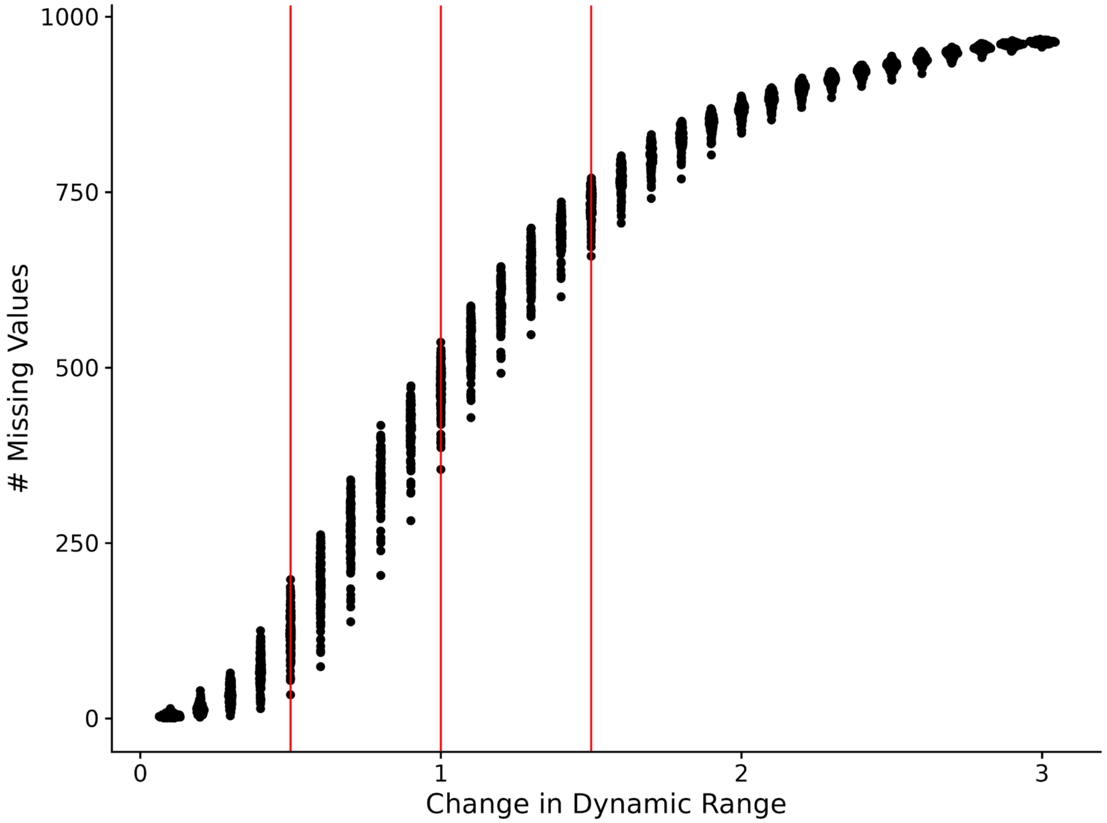

Figure S12. Number of missing values in each of the 100 samples as a function of changing the dynamic range of the values in the sample by increasing the lower limit of detection.

bioRxiv preprint doi: https://doi.org/10.1101/2022.02.24.481854; this version posted July 21, 2025. The copyright holder for this preprint (which was not certified by peer review) is the author/funder, who has granted bioRxiv a license to display the preprint in perpetuity. It is made available under aCC-BY 4.0 International license.

Performance

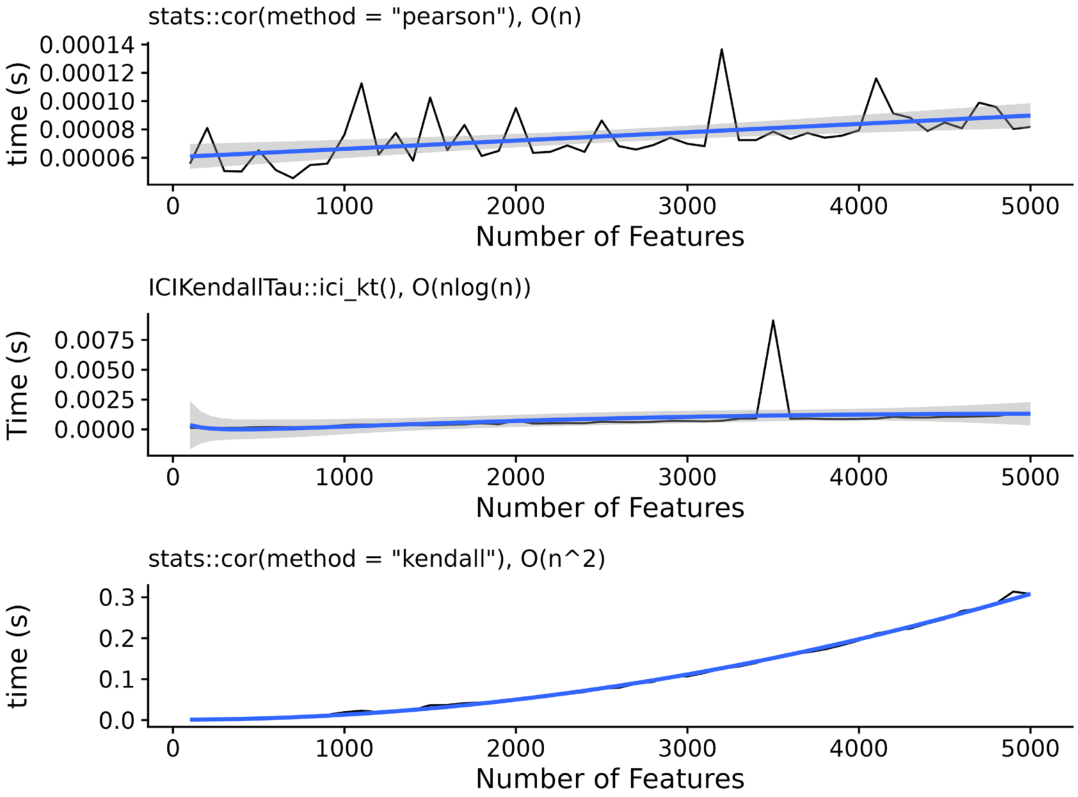

Figure S13. Time in seconds needed as a function of the number of features, with a fitted line for the assumed complexity for each of the methods tested, including R's Pearson correlation, the ICI-Kt mergesort, and R's Kendall-tau correlation algorithm.

bioRxiv preprint doi: https://doi.org/10.1101/2022.02.24.481854; this version posted July 21, 2025. The copyright holder for this preprint (which was not certified by peer review) is the author/funder, who has granted bioRxiv a license to display the preprint in perpetuity. It is made available under aCC-BY 4.0 International license.

Outlier Samples

Gierlinski Yeast

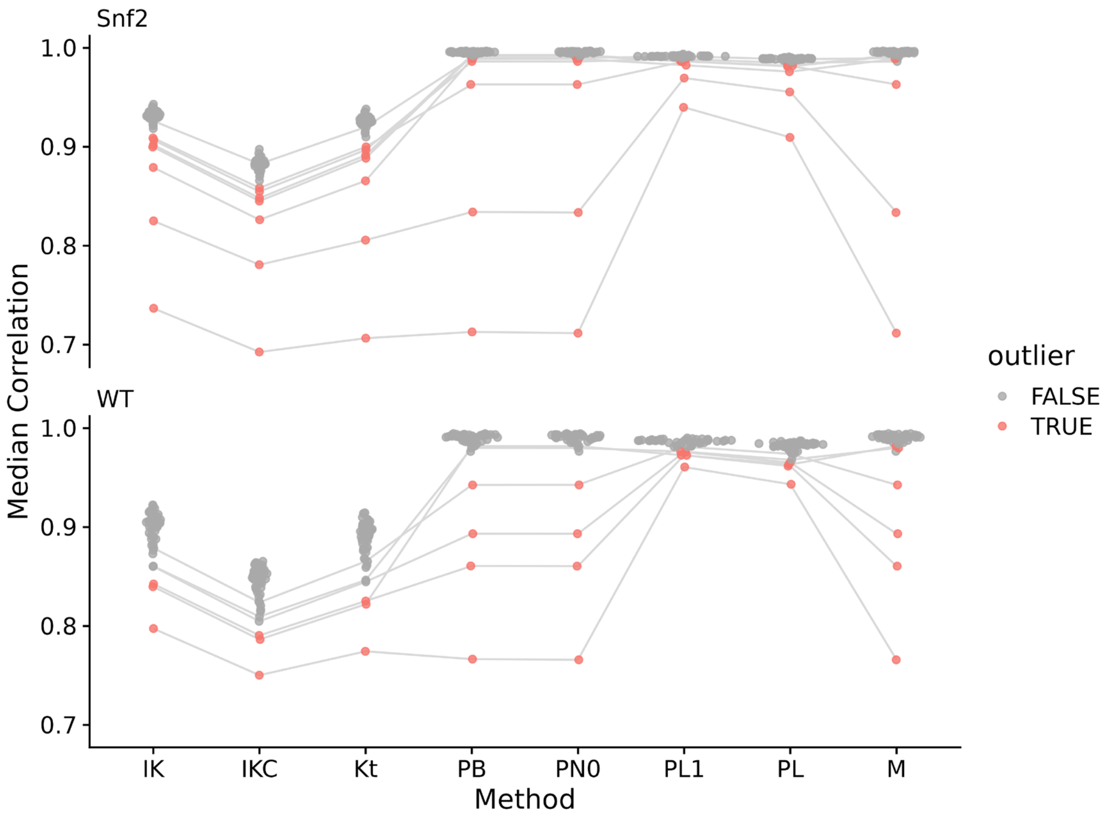

Figure S14. Median correlations for each of the yeast RNA-Seq samples to all other samples in the same group, using different correlation measures on different data. Abbreviations for different measures and data are: IK: ICI-Kt; IKC: ICI-Kt * Completeness; M: Manuscript; PL1: Pearson Log(x + 1); PN0: Pearson No Zeros. Points are colored red if they were an outlier using that correlation method. For a sample that is considered an outlier in any of the method, lines are drawn connecting them between methods.

Table S1. Yeast dataset median correlation values and outlier determination for each outlier from each of the correlation methods. Abbreviations for different measures are: IK: ICI-Kt; IKC: ICI-Kt * Completeness; PB: Pearson Base, PN: Pearson No Zero; PL1: Pearson Log(x + 1); PL: Pearson Log(x); Kt: Kendall-tau.

Sample	IK	IKC	Kt	PB	PN0	PL1	PL	M
Snf2.31	**0.902**	**0.848**	**0.891**	0.993	0.993	**0.987**	**0.982**	0.993
Snf2.15	**0.900**	**0.845**	**0.888**	0.992	0.992	**0.986**	**0.981**	0.992
Snf2.24	0.926	0.883	0.920	**0.990**	**0.990**	0.991	0.988	0.990

bioRxiv preprint doi: https://doi.org/10.1101/2022.02.24.481854; this version posted July 21, 2025. The copyright holder for this preprint (which was not certified by peer review) is the author/funder, who has granted bioRxiv a license to display the preprint in perpetuity. It is made available under aCC-BY 4.0 International license.

Sample	IK	IKC	Kt	PB	PN0	PL1	PL	M
Snf2.25	**0.879**	**0.826**	**0.866**	**0.989**	**0.989**	**0.982**	**0.976**	**0.989**
Snf2.10	**0.907**	**0.855**	**0.897**	**0.986**	**0.986**	**0.989**	0.985	0.986
Snf2.35	**0.909**	**0.858**	**0.900**	**0.963**	**0.963**	**0.986**	**0.982**	**0.963**
Snf2.13	**0.825**	**0.781**	**0.806**	**0.834**	**0.834**	**0.970**	**0.955**	**0.834**
Snf2.06	**0.737**	**0.693**	**0.707**	**0.713**	**0.712**	**0.940**	**0.909**	**0.712**
WT.34	**0.840**	**0.786**	**0.822**	0.982	0.982	**0.973**	**0.963**	**0.982**
WT.36	0.860	0.810	0.846	0.980	0.980	**0.976**	0.968	**0.980**
WT.22	0.879	0.824	0.865	**0.943**	**0.943**	0.981	0.974	**0.943**
WT.28	0.860	0.805	0.845	**0.893**	**0.893**	**0.976**	**0.965**	**0.893**
WT.25	**0.843**	**0.790**	**0.825**	**0.861**	**0.861**	**0.973**	**0.962**	**0.861**
WT.21	**0.797**	**0.750**	**0.774**	**0.767**	**0.766**	**0.961**	**0.943**	**0.766**

EGFR RNASeq Genotype

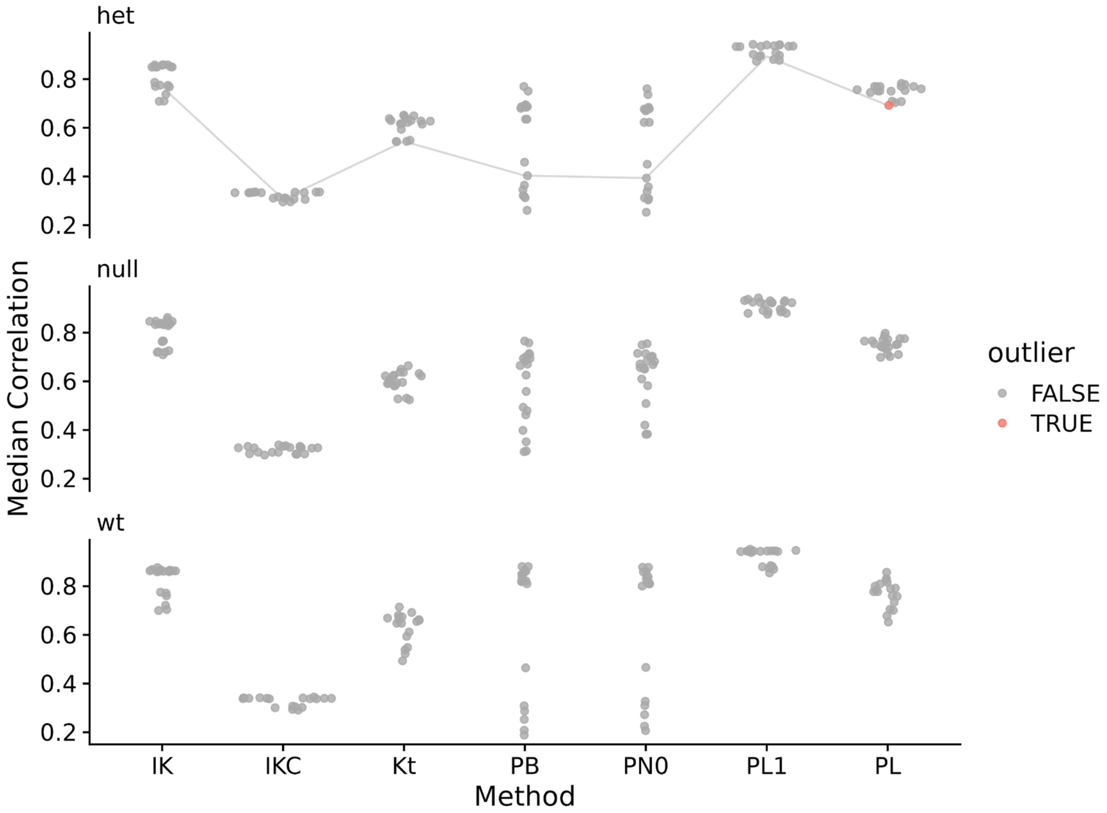

bioRxiv preprint doi: https://doi.org/10.1101/2022.02.24.481854; this version posted July 21, 2025. The copyright holder for this preprint (which was not certified by peer review) is the author/funder, who has granted bioRxiv a license to display the preprint in perpetuity. It is made available under aCC-BY 4.0 International license.

Figure S15. Median correlations for each of the Brainson RNA-Seq samples to all other samples in the same group, using different correlation measures on different data. Abbreviations for different measures and data are: IK: ICI-Kt; IKC: ICI-Kt * Completeness; PB: Pearson Base, PN: Pearson No Zero; PL1: Pearson Log(x + 1); PL: Pearson Log(x); Kt: Kendall-tau. Points are colored red if they were an outlier using that correlation method. For a sample that is considered an outlier in any of the method, lines are drawn connecting them between methods.

Table S2. EGFR RNA-Seq dataset using genotype as the grouping variable median correlation values and outlier determination for each outlier from each of the correlation methods. Abbreviations for different measures and data are: IK: ICI-Kt; IKC: ICI-Kt * Completeness; PB: Pearson Base, PN: Pearson No Zero; PL1: Pearson Log(x + 1); PL: Pearson Log(x); Kt: Kendall-tau.

Sample	IK	IKC	Kt	PB	PN0	PL1	PL
783-het.sorted	0.768	0.307	0.544	0.403	0.394	0.893	**0.692**

TCGA Adenocarcinoma

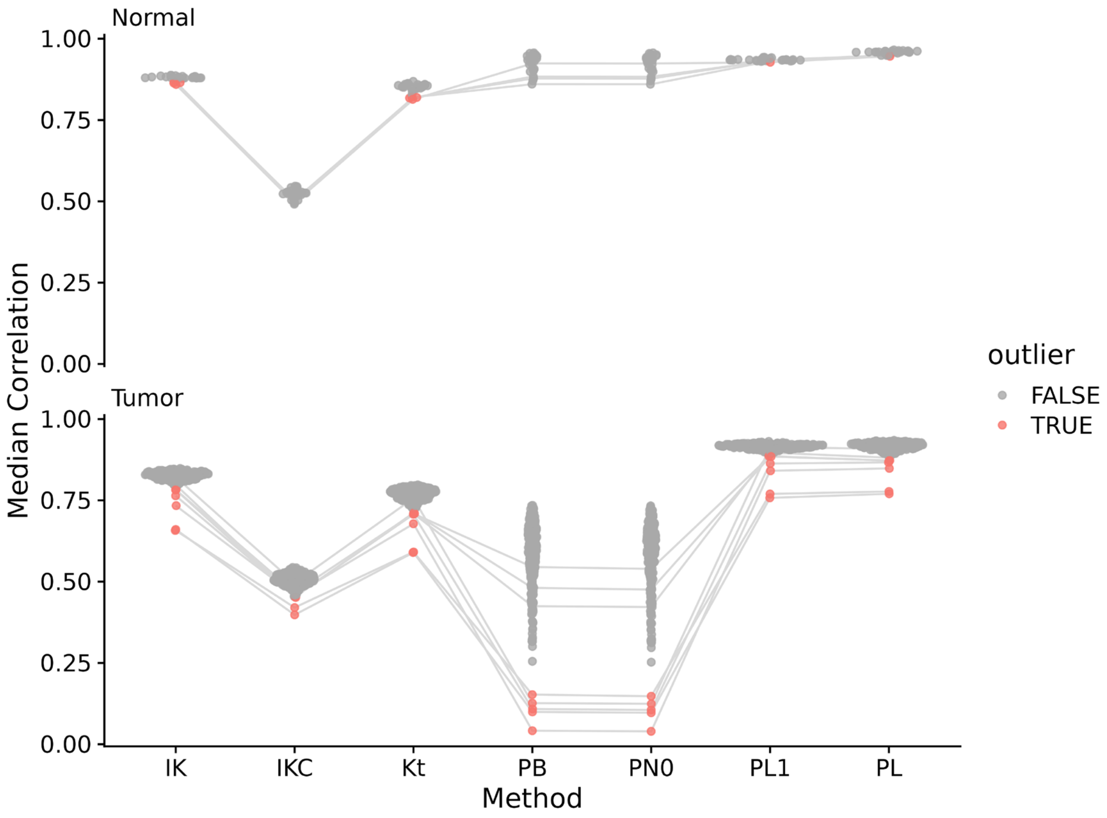

Figure S16. Median correlations for each of the TGCA adenocarcinoma RNA-Seq samples to all other samples in the same group, using different correlation measures on different data. Abbreviations for different measures and data are: IK: ICI-Kt; IKC: ICI-Kt *

bioRxiv preprint doi: https://doi.org/10.1101/2022.02.24.481854; this version posted July 21, 2025. The copyright holder for this preprint (which was not certified by peer review) is the author/funder, who has granted bioRxiv a license to display the preprint in perpetuity. It is made available under aCC-BY 4.0 International license.

Completeness; PB: Pearson Base, PN: Pearson No Zero; PL1: Pearson Log(x + 1); PL: Pearson Log(x); Kt: Kendall-tau. Points are colored red if they were an outlier using that correlation method. For a sample that is considered an outlier in any of the method, lines are drawn connecting them between methods.

Table S3. TGCA adenocarcinoma dataset median correlation values and outlier determination for each outlier from each of the correlation methods. Abbreviations for different measures and data are: IK: ICI-Kt; IKC: ICI-Kt * Completeness; PB: Pearson Base, PN: Pearson No Zero; PL1: Pearson Log(x + 1); PL: Pearson Log(x); Kt: Kendall-tau.

Sample	IK	IKC	Kt	PB	PN0	PL1	PL
N.24	**0.864**	0.504	**0.819**	0.878	0.877	0.934	0.949
N.13	**0.865**	0.504	**0.818**	0.883	0.883	0.933	0.949
N.18	**0.867**	0.499	**0.820**	0.860	0.860	0.932	0.948
N.22	**0.859**	0.495	**0.814**	0.924	0.924	**0.928**	**0.946**
T.39	0.821	0.496	0.757	**0.126**	**0.124**	0.914	0.908
T.122	0.798	**0.453**	**0.710**	0.425	0.422	0.897	0.881
T.180	**0.783**	0.467	**0.709**	0.545	0.539	**0.886**	**0.872**
T.220	**0.782**	**0.452**	**0.712**	0.481	0.475	**0.885**	**0.872**
T.38	**0.764**	0.454	**0.707**	**0.109**	**0.105**	**0.863**	**0.867**
T.261	**0.734**	0.459	**0.678**	**0.042**	**0.040**	**0.841**	**0.848**
T.40	**0.657**	**0.420**	**0.591**	**0.153**	**0.148**	**0.770**	**0.777**
T.260	**0.661**	**0.398**	**0.590**	**0.099**	**0.097**	**0.758**	**0.770**

Table S4. Adenocarcinoma median-absolute-deviations (MAD) for each group of sample median correlations in each type of tissue.

method	Normal	Tumor
IK	0.004	0.012
IKC	0.011	0.019
Kt	0.008	0.019
PB	0.022	0.092
PN0	0.021	0.091
PL1	0.002	0.008

bioRxiv preprint doi: https://doi.org/10.1101/2022.02.24.481854; this version posted July 21, 2025. The copyright holder for this preprint (which was not certified by peer review) is the author/funder, who has granted bioRxiv a license to display the preprint in perpetuity. It is made available under aCC-BY 4.0 International license.

method	Normal	Tumor
PL	0.003	0.012

NSCLC Lipidomics

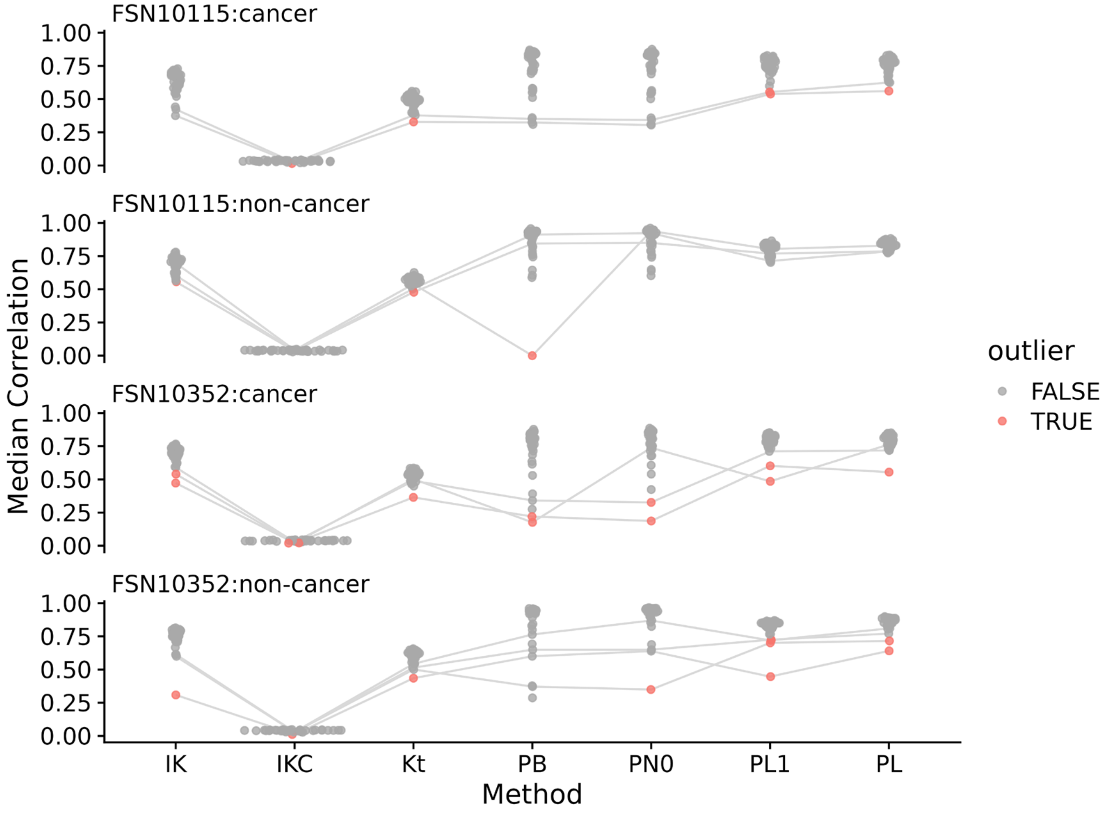

Figure S17. Median correlations for each of the NSCLC lipidomics samples to all other samples in the same group, using different correlation measures on different data. Abbreviations for different measures and data are: IK: ICI-Kt; IKC: ICI-Kt * Completeness; PB: Pearson Base, PN: Pearson No Zero; PL1: Pearson Log(x + 1); PL: Pearson Log(x); Kt: Kendall-tau. Points are colored red if they were an outlier using that correlation method. For a sample that is considered an outlier in any of the method, lines are drawn connecting them between methods.

Table S5. NSCLC lipidomics dataset median correlation values and outlier determination for each outlier from each of the correlation methods. Abbreviations for different measures and data are: IK: ICI-Kt; IKC: ICI-Kt * Completeness; PB: Pearson Base, PN: Pearson No Zero; PL1: Pearson Log(x + 1); PL: Pearson Log(x); Kt: Kendall-tau.

Sample	IK	IKC	Kt	PB	PN0	PL1	PL
172Cpos	0.423	0.021	0.379	0.351	0.342	**0.553**	0.625

bioRxiv preprint doi: https://doi.org/10.1101/2022.02.24.481854; this version posted July 21, 2025. The copyright holder for this preprint (which was not certified by peer review) is the author/funder, who has granted bioRxiv a license to display the preprint in perpetuity. It is made available under aCC-BY 4.0 International license.

Sample	IK	IKC	Kt	PB	PN0	PL1	PL
73Cpos	0.375	**0.015**	**0.328**	0.324	0.305	**0.538**	**0.561**
73Npos	0.698	0.041	0.539	**-0.000**	0.939	0.803	0.830
78Npos	0.604	0.036	**0.478**	0.845	0.849	0.769	0.786
150Npos	**0.557**	0.034	0.504	0.911	0.923	0.713	0.786
205Cpos	0.591	0.028	0.490	0.341	**0.327**	0.711	0.718
uk003cposb	**0.540**	**0.020**	**0.365**	**0.220**	**0.187**	**0.602**	**0.555**
47Cpos	**0.472**	**0.021**	0.503	**0.176**	0.738	**0.485**	0.763
155Nposr	0.600	0.028	0.515	0.650	0.650	**0.724**	0.771
201Npos	0.612	0.030	0.543	0.764	0.870	**0.720**	0.810
169Npos	0.615	0.029	0.501	0.372	**0.350**	**0.701**	**0.716**
148Npos	**0.309**	**0.011**	**0.435**	0.600	0.639	**0.447**	**0.641**

Rat Stamina Metabolomics

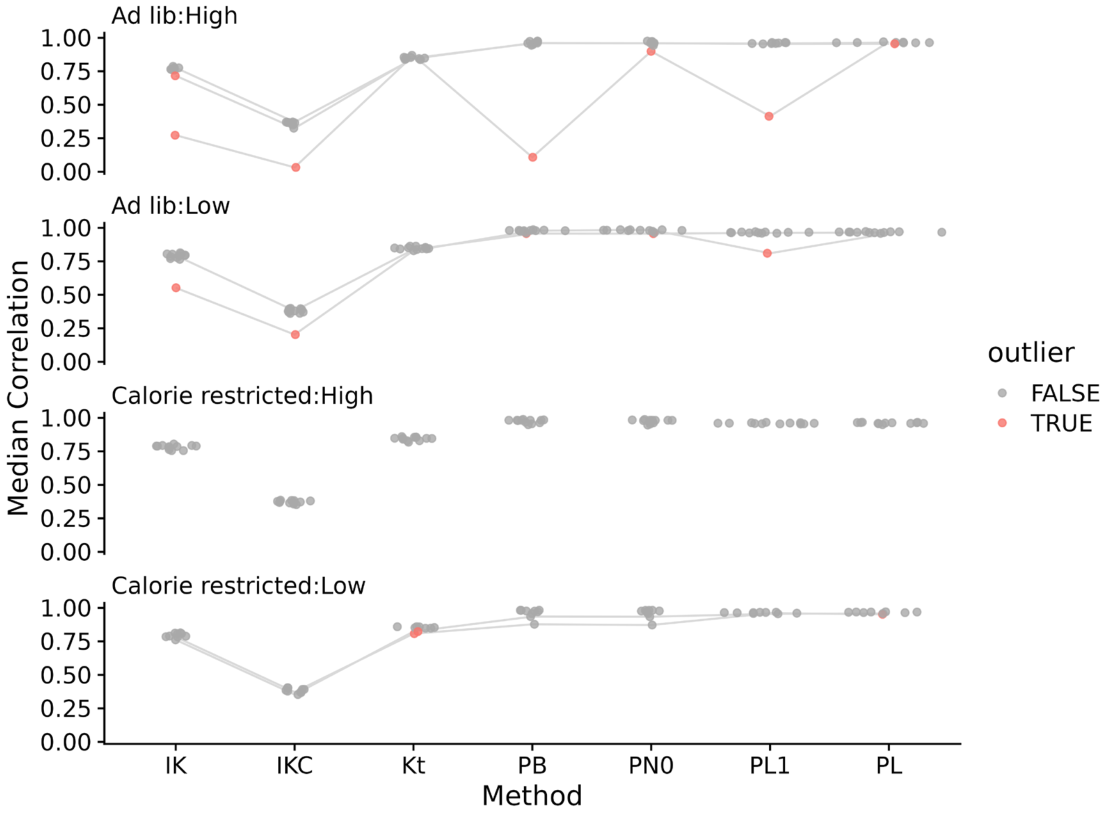

bioRxiv preprint doi: https://doi.org/10.1101/2022.02.24.481854; this version posted July 21, 2025. The copyright holder for this preprint (which was not certified by peer review) is the author/funder, who has granted bioRxiv a license to display the preprint in perpetuity. It is made available under aCC-BY 4.0 International license.

Figure S18. Median correlations for each of the rat stamina metabolomics samples to all other samples in the same group, using different correlation measures on different data. Abbreviations for different measures and data are: IK: ICI-Kt; IKC: ICI-Kt * Completeness; PB: Pearson Base, PN: Pearson No Zero; PL1: Pearson Log(x + 1); PL: Pearson Log(x); Kt: Kendall-tau. Points are colored red if they were an outlier using that correlation method. For a sample that is considered an outlier in any of the method, lines are drawn connecting them between methods.

Table S6. Rat stamina metabolomics dataset median correlation values and outlier determination for each outlier from each of the correlation methods. Abbreviations for different measures and data are: IK: ICI-Kt; IKC: ICI-Kt * Completeness; PB: Pearson Base, PN: Pearson No Zero; PL1: Pearson Log(x + 1); PL: Pearson Log(x); Kt: Kendall-tau.

Sample	IK	IKC	Kt	PB	PN0	PL1	PL
s00009502	**0.717**	0.325	0.848	0.959	0.959	0.957	0.963
s00009501	0.776	0.371	0.844	0.961	0.960	0.955	**0.955**
s00009481	**0.273**	**0.032**	0.869	**0.108**	**0.899**	**0.414**	0.970
s00009471	0.794	0.380	0.838	**0.957**	**0.957**	0.962	0.965
s00009470	**0.553**	**0.203**	0.829	0.979	0.985	**0.810**	0.959
s00009484	0.783	0.372	**0.808**	0.878	0.872	0.961	**0.953**
s00009508	0.762	0.353	**0.823**	0.935	0.934	0.956	0.959

bioRxiv preprint doi: https://doi.org/10.1101/2022.02.24.481854; this version posted July 21, 2025. The copyright holder for this preprint (which was not certified by peer review) is the author/funder, who has granted bioRxiv a license to display the preprint in perpetuity. It is made available under aCC-BY 4.0 International license.

Effect of Increasing Presence in Samples

Yeast Samples

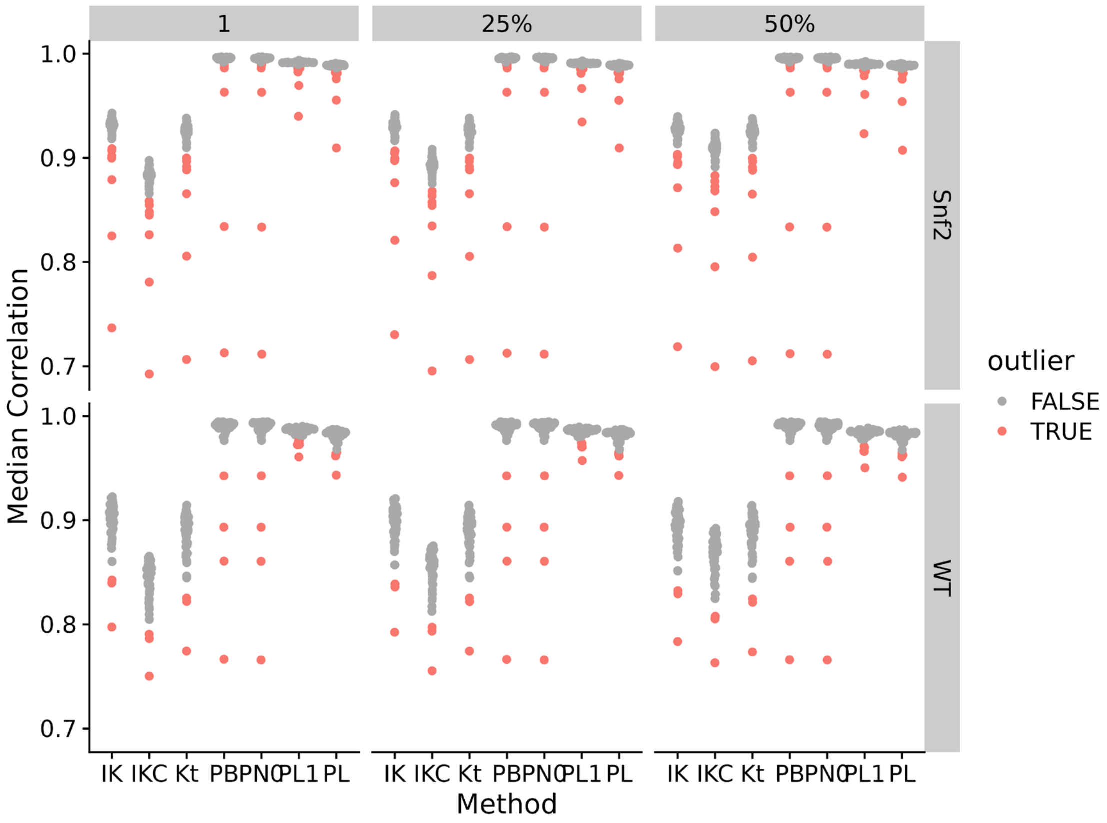

Figure S19. Median correlations by correlation method and applying different fractional cutoffs. Abbreviations for different measures and data are: IK: ICI-Kt; IKC: ICI-Kt * Completeness; Kt: Kendall-tau; PB: Pearson Base (raw values); PL: Pearson Log(x); PL1: Pearson Log(x + 1); PN0: Pearson No Zeros.

bioRxiv preprint doi: https://doi.org/10.1101/2022.02.24.481854; this version posted July 21, 2025. The copyright holder for this preprint (which was not certified by peer review) is the author/funder, who has granted bioRxiv a license to display the preprint in perpetuity. It is made available under aCC-BY 4.0 International license.

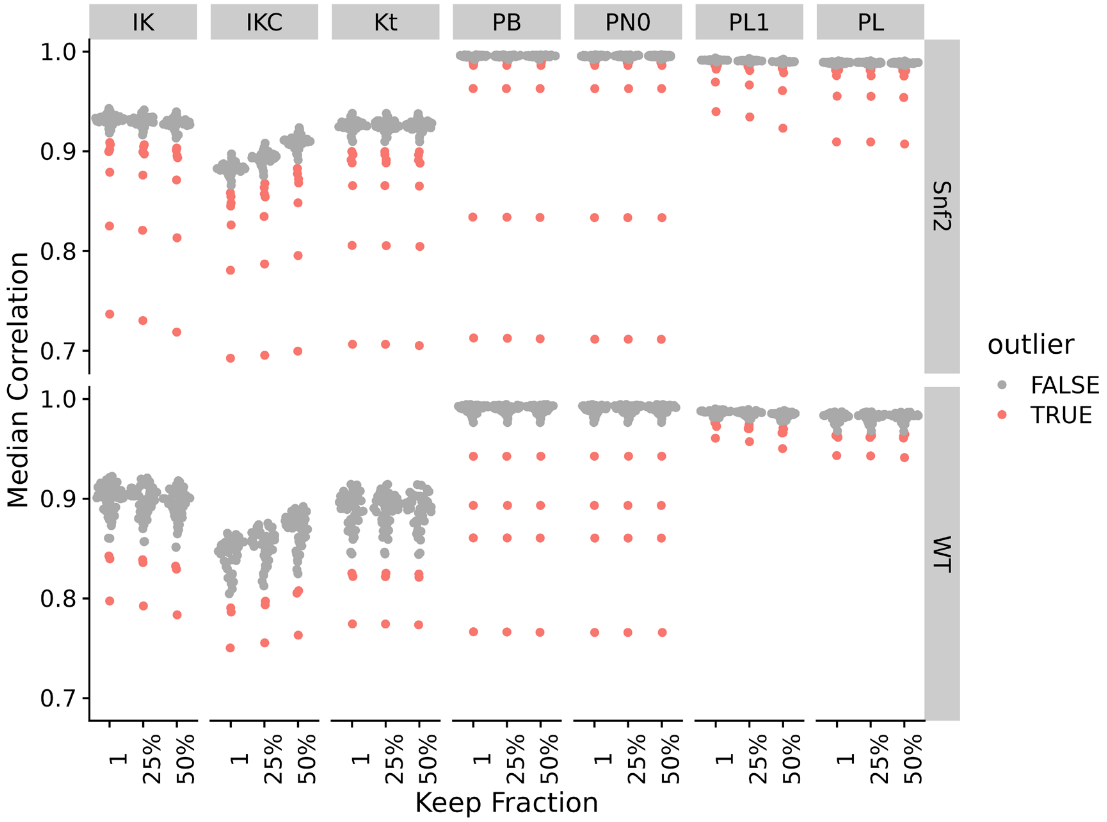

Figure S20. Median correlations by applying different fractional cutoffs and different correlation methods. Abbreviations for different measures and data are: IK: ICI-Kt; IKC: ICI-Kt * Completeness; Kt: Kendall-tau; PB: Pearson Base (raw values); PL: Pearson Log(x); PL1: Pearson Log(x + 1); PN0: Pearson No Zeros.

bioRxiv preprint doi: https://doi.org/10.1101/2022.02.24.481854; this version posted July 21, 2025. The copyright holder for this preprint (which was not certified by peer review) is the author/funder, who has granted bioRxiv a license to display the preprint in perpetuity. It is made available under aCC-BY 4.0 International license.

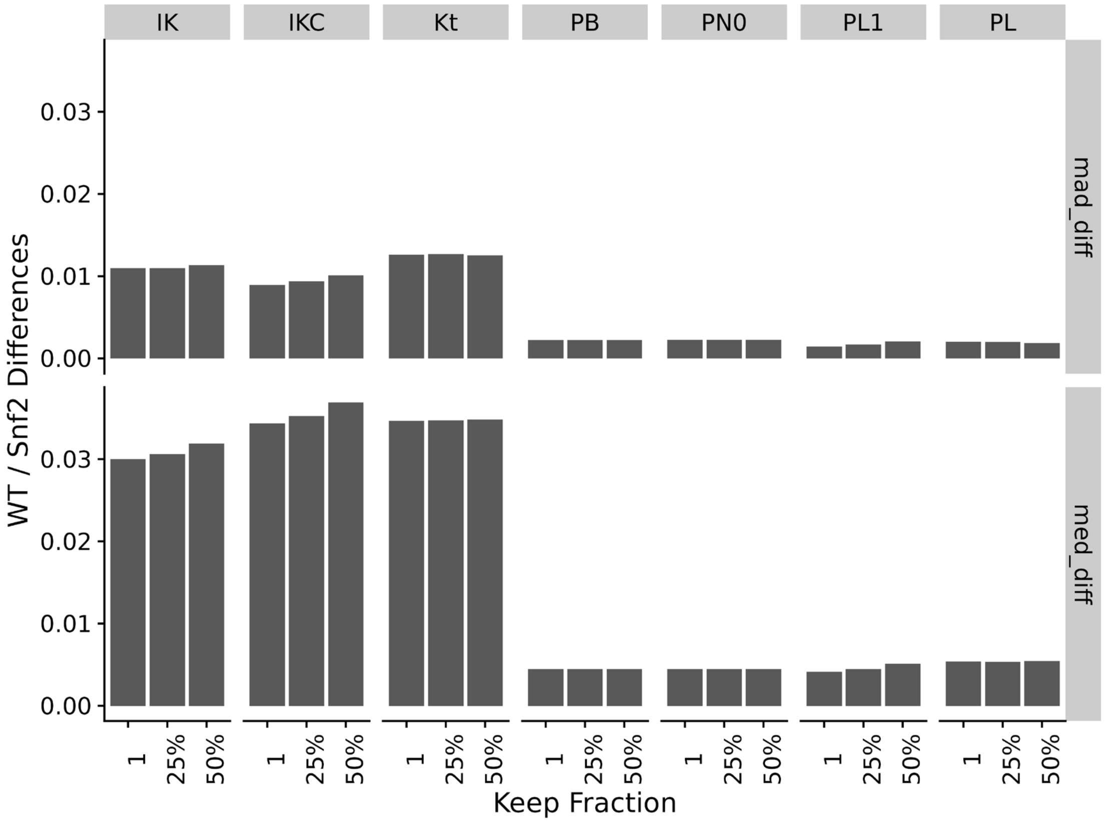

Figure S21. Difference between WT and Snf2 group medians and MAD for the different correlation methods and gene presence fractions. Abbreviations for different measures and data are: IK: ICI-Kt; IKC: ICI-Kt * Completeness; Kt: Kendall-tau; PB: Pearson Base (raw values); PL: Pearson Log(x); PL1: Pearson Log(x + 1); PN0: Pearson No Zeros.

bioRxiv preprint doi: https://doi.org/10.1101/2022.02.24.481854; this version posted July 21, 2025. The copyright holder for this preprint (which was not certified by peer review) is the author/funder, who has granted bioRxiv a license to display the preprint in perpetuity. It is made available under aCC-BY 4.0 International license.

EGFR Genotype Samples

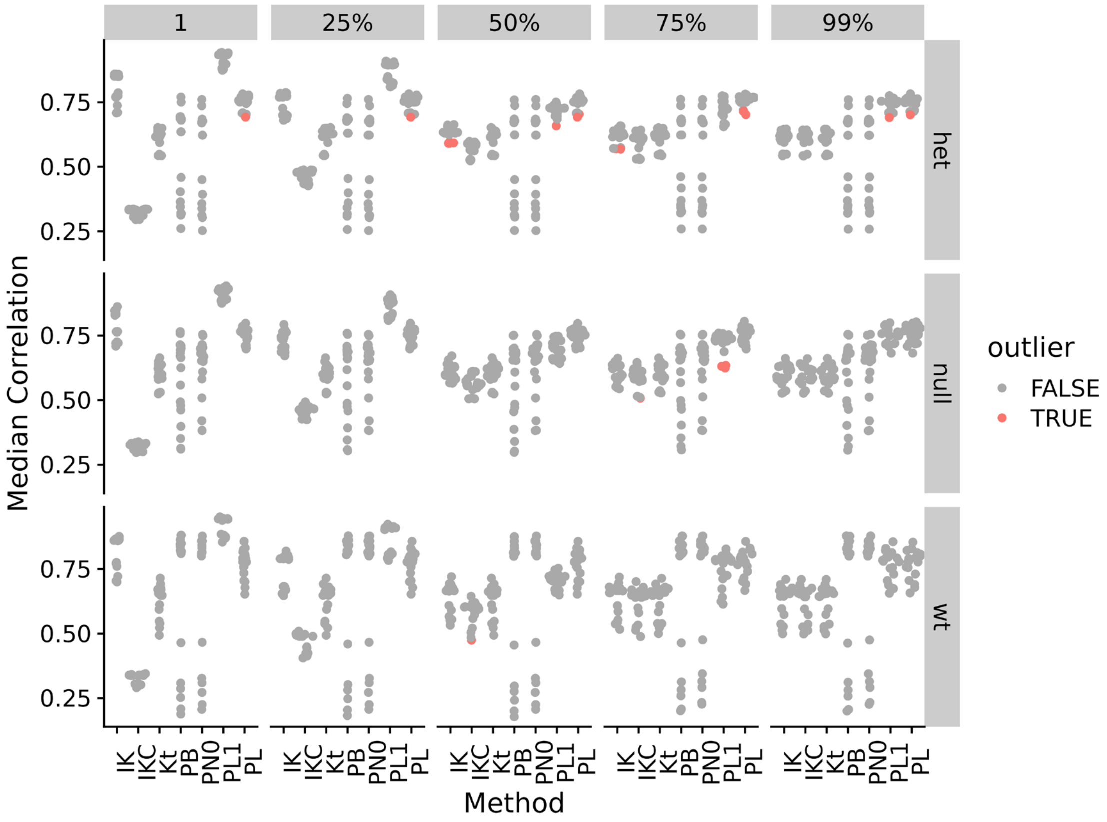

Figure S22. Median correlations by correlation method and applying different fractional cutoffs for Brainson RNA-seq data. Abbreviations for different measures and data are: IK: ICI-Kt; IKC: ICI-Kt * Completeness; Kt: Kendall-tau; PB: Pearson Base (raw values); PL: Pearson Log(x); PL1: Pearson Log(x + 1); PN0: Pearson No Zeros.

bioRxiv preprint doi: https://doi.org/10.1101/2022.02.24.481854; this version posted July 21, 2025. The copyright holder for this preprint (which was not certified by peer review) is the author/funder, who has granted bioRxiv a license to display the preprint in perpetuity. It is made available under aCC-BY 4.0 International license.

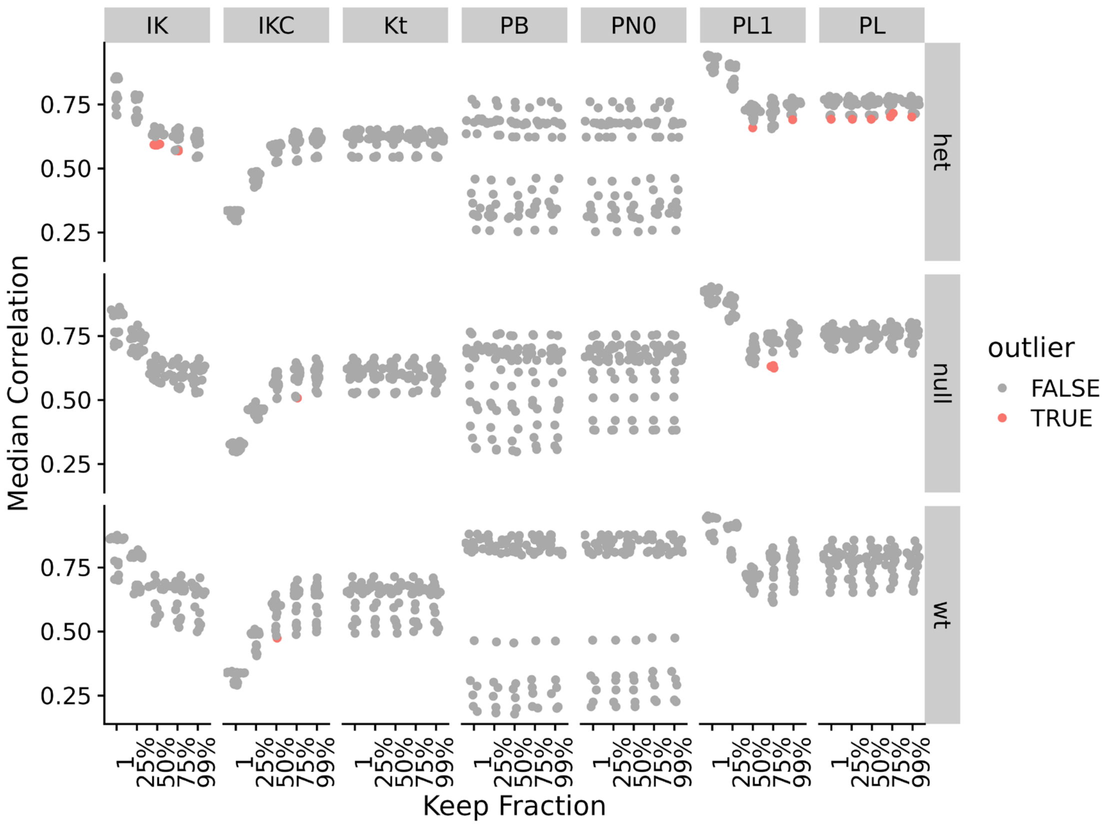

Figure S23. Median correlations by correlation method and applying different fractional cutoffs for Brainson RNA-seq data. Abbreviations for different measures and data are: IK: ICI-Kt; IKC: ICI-Kt * Completeness; Kt: Kendall-tau; PB: Pearson Base (raw values); PL: Pearson Log(x); PL1: Pearson Log(x + 1); PN0: Pearson No Zeros.

bioRxiv preprint doi: https://doi.org/10.1101/2022.02.24.481854; this version posted July 21, 2025. The copyright holder for this preprint (which was not certified by peer review) is the author/funder, who has granted bioRxiv a license to display the preprint in perpetuity. It is made available under aCC-BY 4.0 International license.

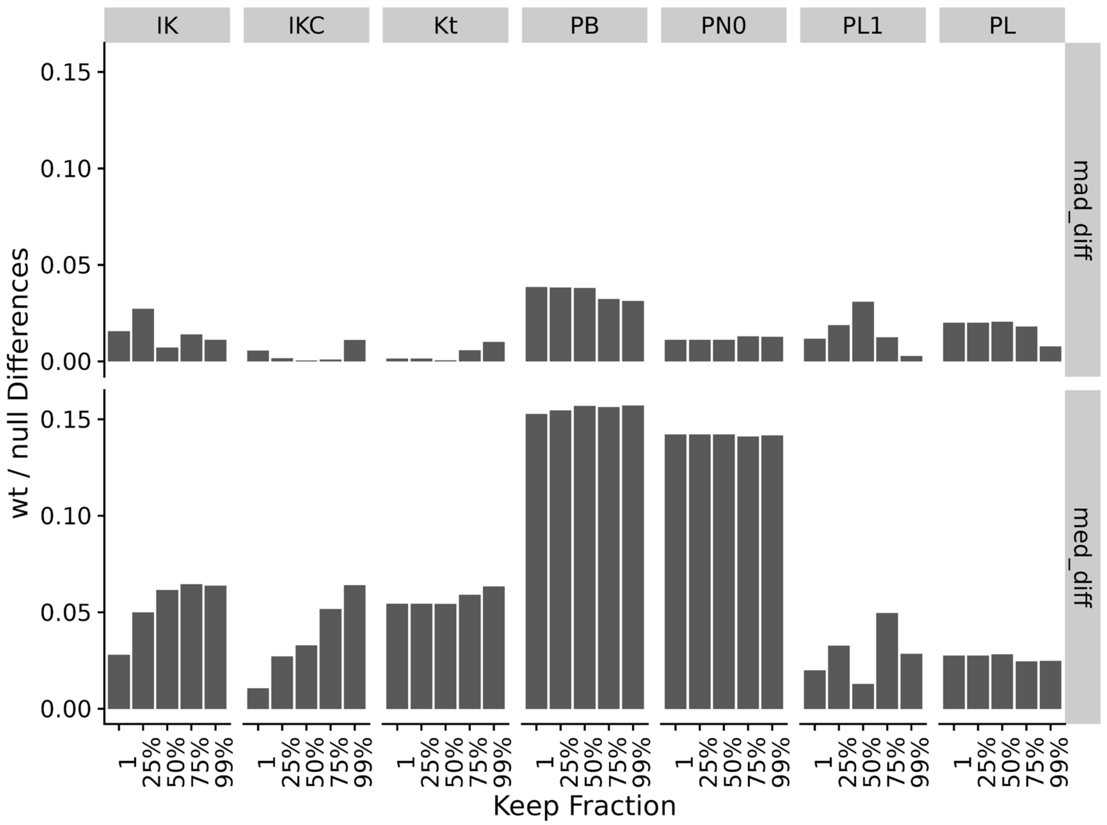

Figure S24. Difference between *wt* and *null* sample group medians and MAD for the different correlation methods and gene presence fractions. Abbreviations for different measures and data are: IK: ICI-Kt; IKC: ICI-Kt * Completeness; Kt: Kendall-tau; PB: Pearson Base (raw values); PL: Pearson Log(x); PL1: Pearson Log(x + 1); PN0: Pearson No Zeros.

bioRxiv preprint doi: https://doi.org/10.1101/2022.02.24.481854; this version posted July 21, 2025. The copyright holder for this preprint (which was not certified by peer review) is the author/funder, who has granted bioRxiv a license to display the preprint in perpetuity. It is made available under aCC-BY 4.0 International license.

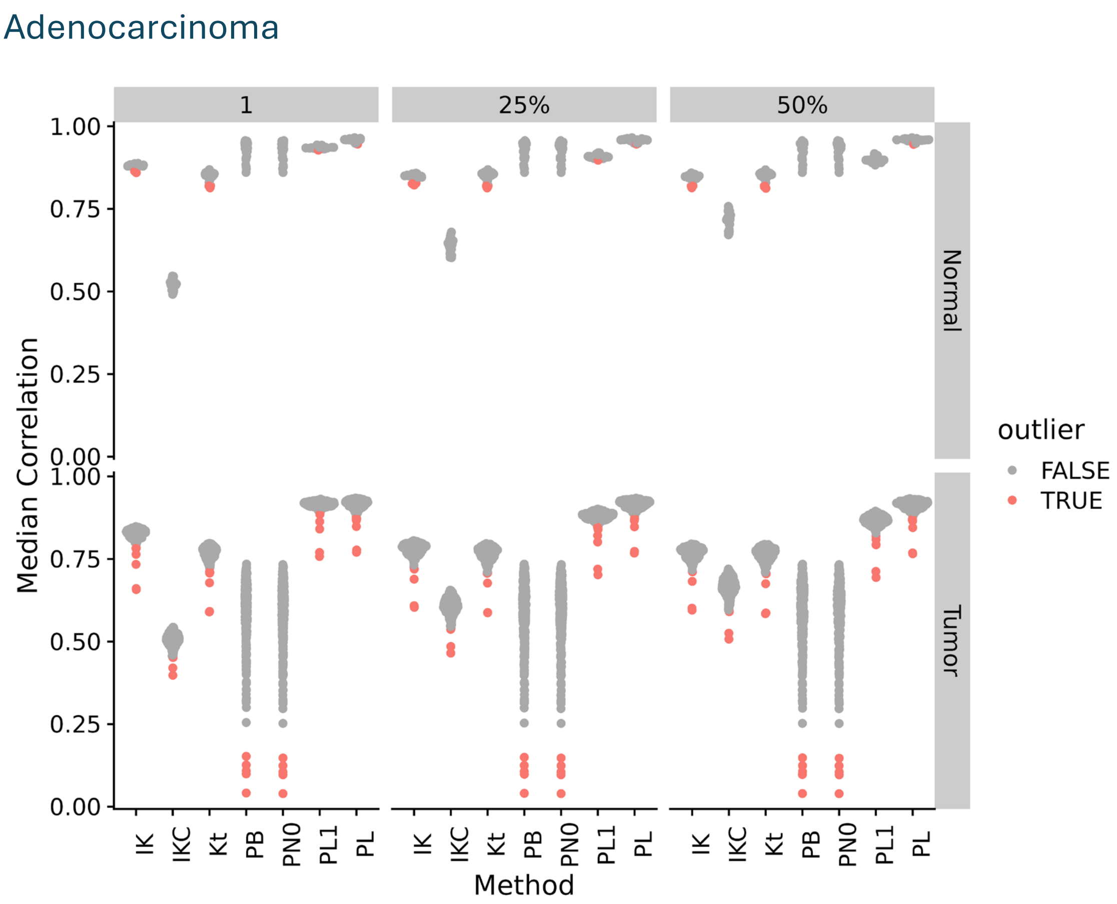

Figure S25. Median correlations by correlation method and applying different fractional cutoffs for TCGA adenocarcinoma RNA-seq data. Abbreviations for different measures and data are: IK: ICI-Kt; IKC: ICI-Kt * Completeness; Kt: Kendall-tau; PB: Pearson Base (raw values); PL: Pearson Log(x); PL1: Pearson Log(x + 1); PN0: Pearson No Zeros.

bioRxiv preprint doi: https://doi.org/10.1101/2022.02.24.481854; this version posted July 21, 2025. The copyright holder for this preprint (which was not certified by peer review) is the author/funder, who has granted bioRxiv a license to display the preprint in perpetuity. It is made available under aCC-BY 4.0 International license.

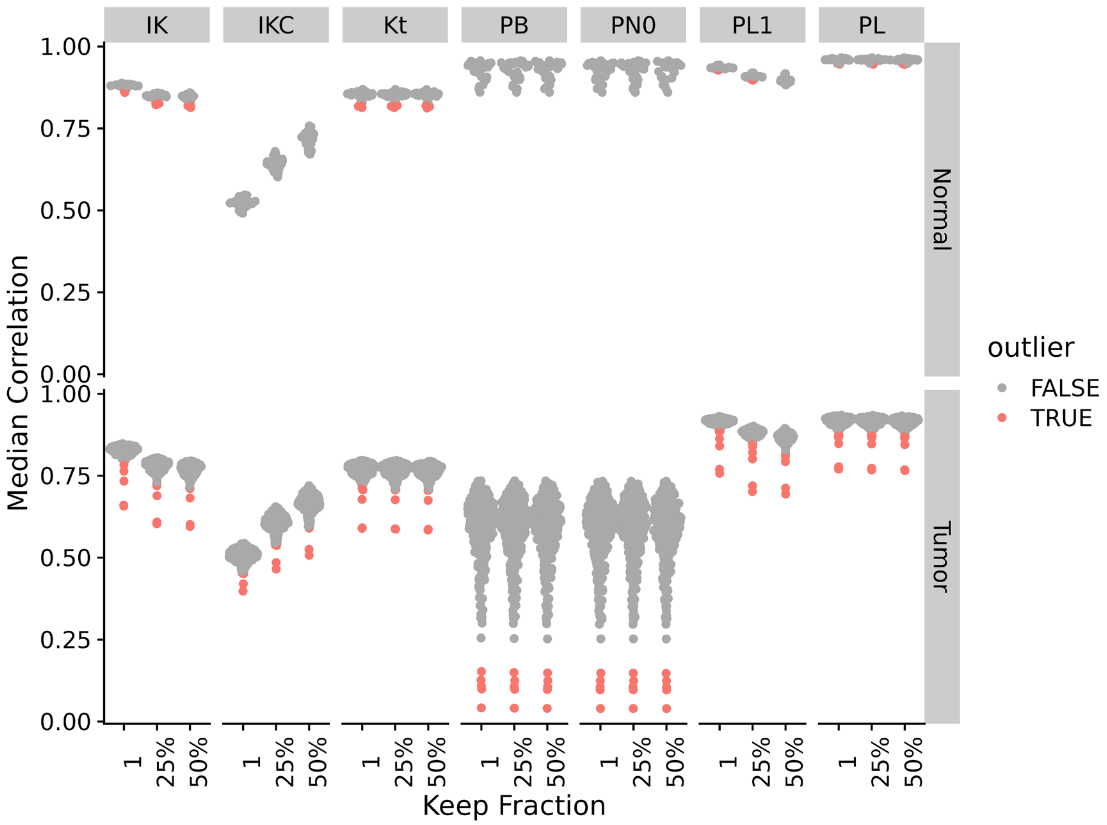

Figure S26. Median correlations by correlation method and applying different fractional cutoffs for TCGA adenocarcinoma RNA-seq data. Abbreviations for different measures and data are: IK: ICI-Kt; IKC: ICI-Kt * Completeness; Kt: Kendall-tau; PB: Pearson Base (raw values); PL: Pearson Log(x); PL1: Pearson Log(x + 1); PN0: Pearson No Zeros.

bioRxiv preprint doi: https://doi.org/10.1101/2022.02.24.481854; this version posted July 21, 2025. The copyright holder for this preprint (which was not certified by peer review) is the author/funder, who has granted bioRxiv a license to display the preprint in perpetuity. It is made available under aCC-BY 4.0 International license.

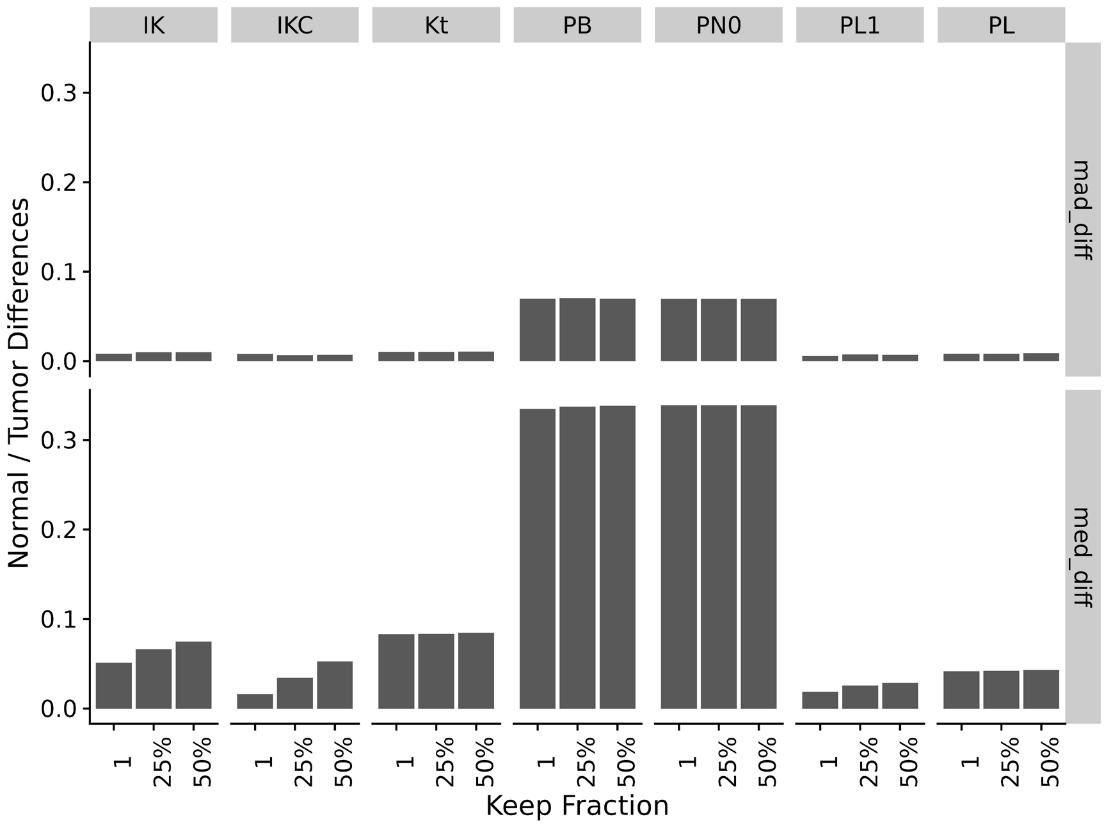

Figure S27. Difference of normal and tumor medians and MADs by correlation method and applying different fractional cutoffs for TCGA adenocarcinoma RNA-seq data. Abbreviations for different measures and data are: IK: ICI-Kt; IKC: ICI-Kt * Completeness; Kt: Kendall-tau; PB: Pearson Base (raw values); PL: Pearson Log(x); PL1: Pearson Log(x + 1); PN0: Pearson No Zeros.

bioRxiv preprint doi: https://doi.org/10.1101/2022.02.24.481854; this version posted July 21, 2025. The copyright holder for this preprint (which was not certified by peer review) is the author/funder, who has granted bioRxiv a license to display the preprint in perpetuity. It is made available under aCC-BY 4.0 International license.

NSCLC

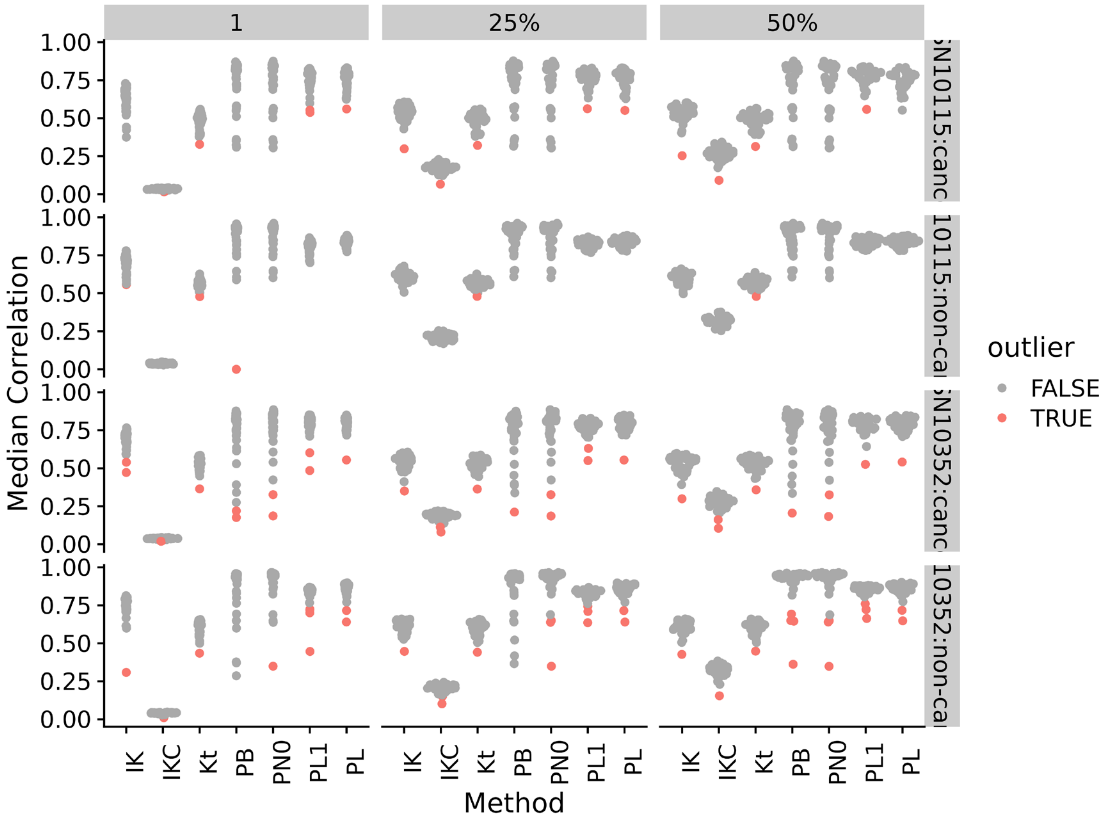

Figure S28. Median correlations by correlation method and applying different fractional cutoffs for NSCLC lipidomics data. Abbreviations for different measures and data are: IK: ICI-Kt; IKC: ICI-Kt * Completeness; Kt: Kendall-tau; PB: Pearson Base (raw values); PL: Pearson Log(x); PL1: Pearson Log(x + 1); PN0: Pearson No Zeros.

bioRxiv preprint doi: https://doi.org/10.1101/2022.02.24.481854; this version posted July 21, 2025. The copyright holder for this preprint (which was not certified by peer review) is the author/funder, who has granted bioRxiv a license to display the preprint in perpetuity. It is made available under aCC-BY 4.0 International license.

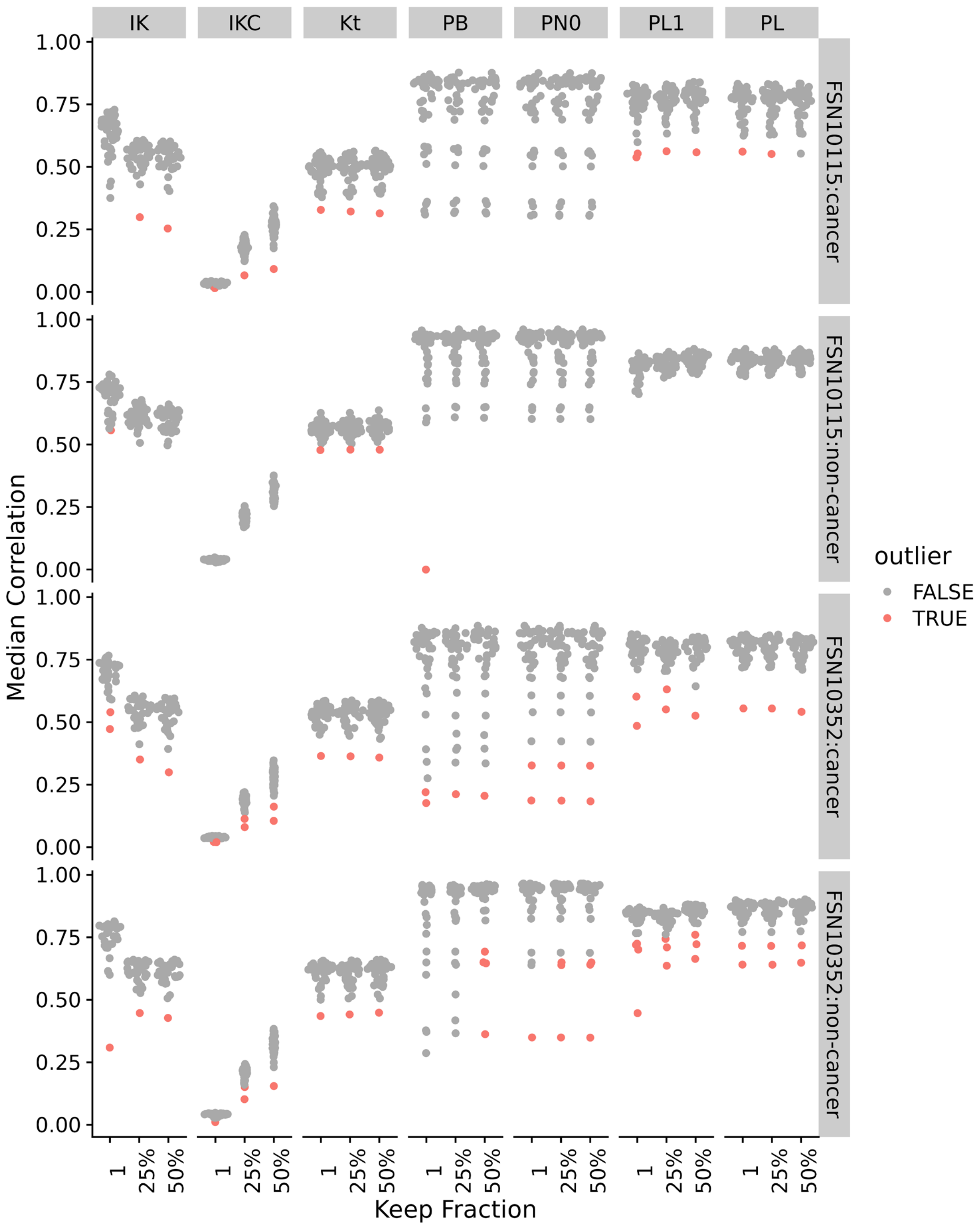

Figure S29. Median correlations by correlation method and applying different fractional cutoffs for NSCLC lipidomics data. Abbreviations for different measures and data are: IK: ICI-Kt; IKC: ICI-Kt * Completeness; Kt: Kendall-tau; PB: Pearson Base (raw values); PL: Pearson Log(x); PL1: Pearson Log(x + 1); PN0: Pearson No Zeros.

bioRxiv preprint doi: https://doi.org/10.1101/2022.02.24.481854; this version posted July 21, 2025. The copyright holder for this preprint (which was not certified by peer review) is the author/funder, who has granted bioRxiv a license to display the preprint in perpetuity. It is made available under aCC-BY 4.0 International license.

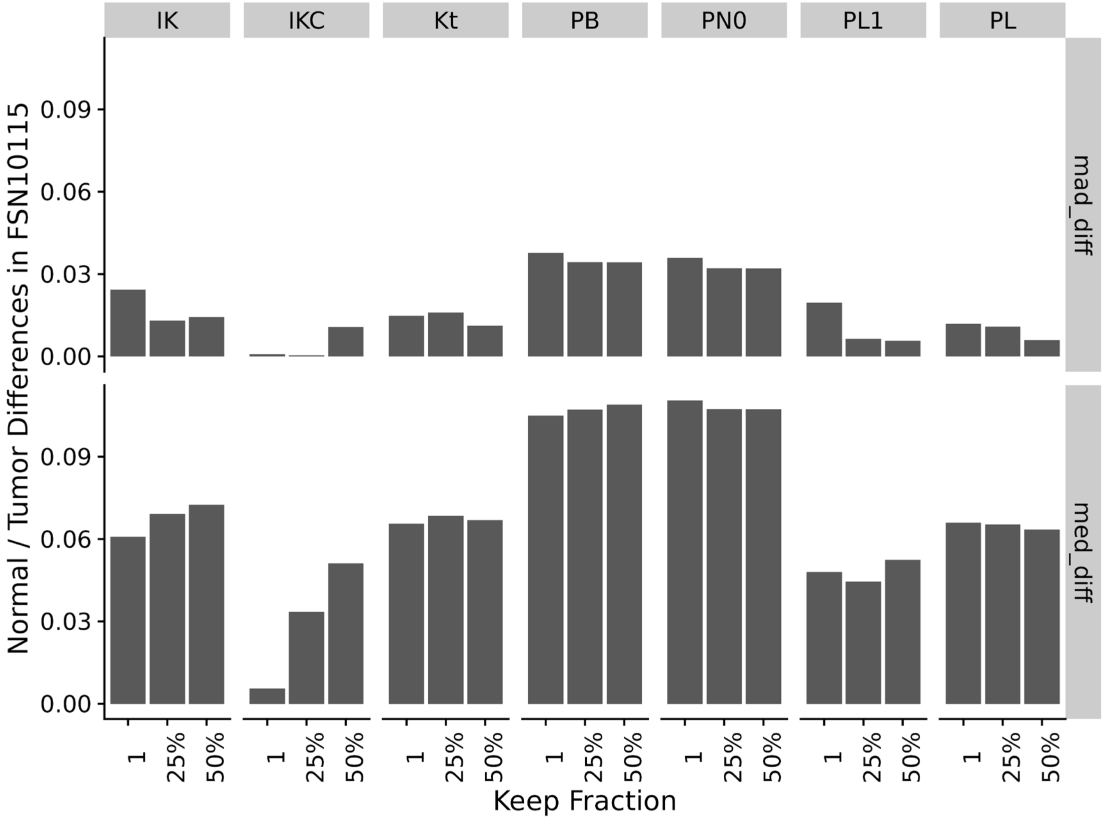

Figure S30. Difference of normal and tumor medians and MADs by correlation method and applying different fractional cutoffs for NSCLC lipidomics data. Abbreviations for different measures and data are: IK: ICI-Kt; IKC: ICI-Kt * Completeness; Kt: Kendall-tau; PB: Pearson Base (raw values); PL: Pearson Log(x); PL1: Pearson Log(x + 1); PN0: Pearson No Zeros.

bioRxiv preprint doi: https://doi.org/10.1101/2022.02.24.481854; this version posted July 21, 2025. The copyright holder for this preprint (which was not certified by peer review) is the author/funder, who has granted bioRxiv a license to display the preprint in perpetuity. It is made available under aCC-BY 4.0 International license.

Rat Stamina

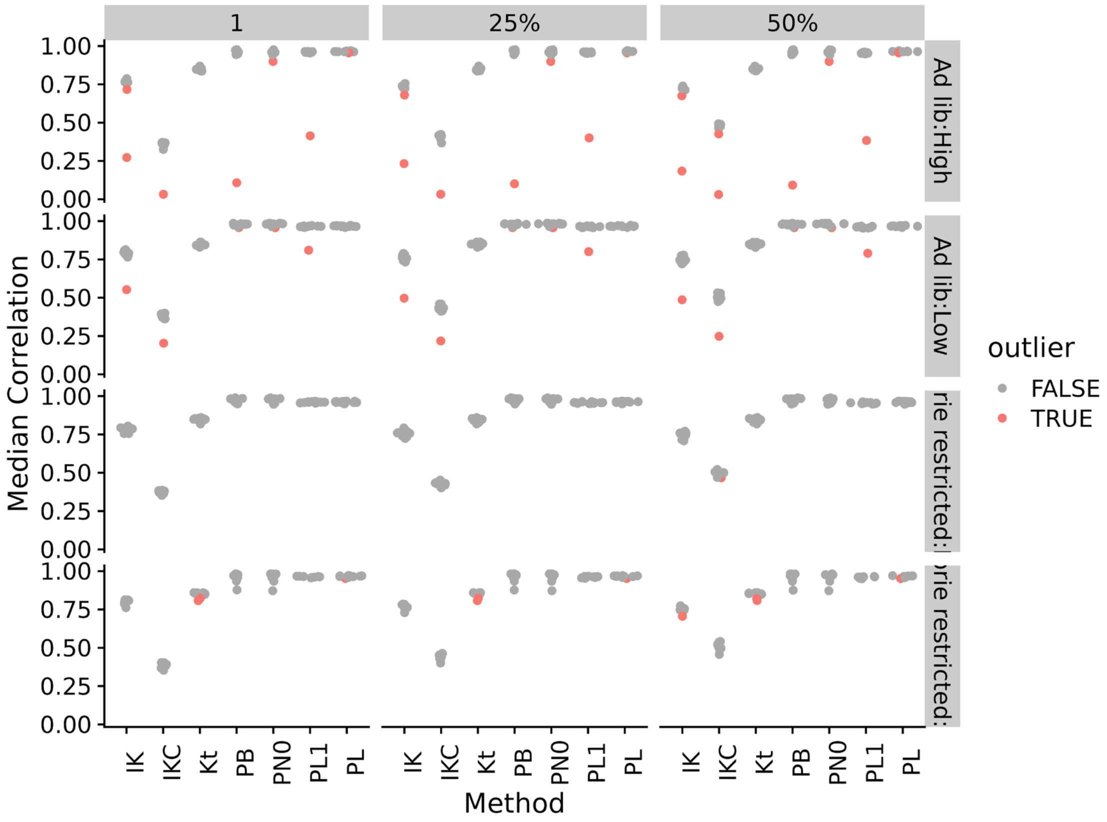

Figure S31. Median correlations by correlation method and applying different fractional cutoffs for rat stamina metabolomics data. Abbreviations for different measures and data are: IK: ICI-Kt; IKC: ICI-Kt * Completeness; Kt: Kendall-tau; PB: Pearson Base (raw values); PL: Pearson Log(x); PL1: Pearson Log(x + 1); PN0: Pearson No Zeros.

bioRxiv preprint doi: https://doi.org/10.1101/2022.02.24.481854; this version posted July 21, 2025. The copyright holder for this preprint (which was not certified by peer review) is the author/funder, who has granted bioRxiv a license to display the preprint in perpetuity. It is made available under aCC-BY 4.0 International license.

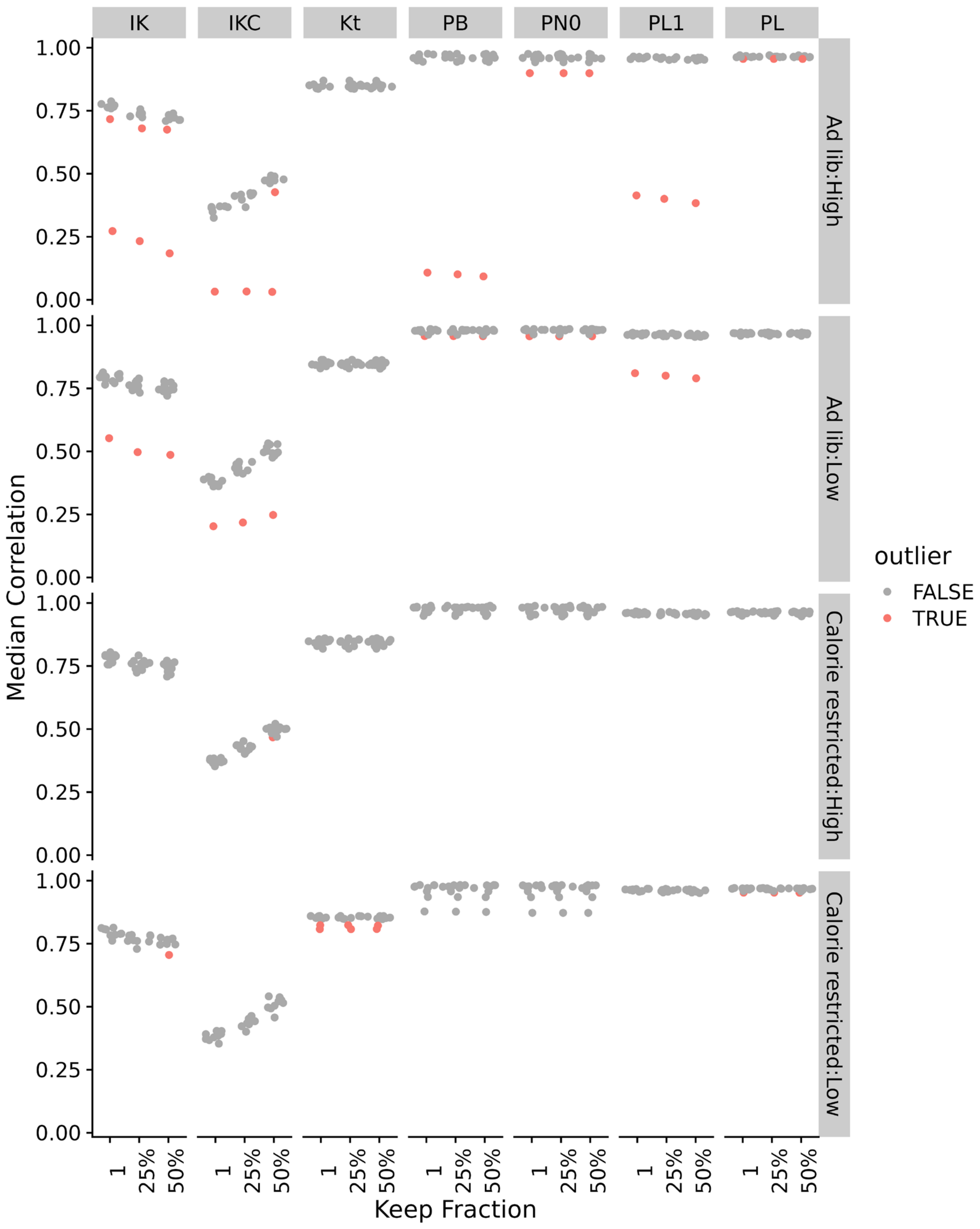

Figure S32. Median correlations by correlation method and applying different fractional cutoffs for rat stamina metabolomics data. Abbreviations for different measures and data are: IK: ICI-Kt; IKC: ICI-Kt * Completeness; Kt: Kendall-tau; PB: Pearson Base (raw values); PL: Pearson Log(x); PL1: Pearson Log(x + 1); PN0: Pearson No Zeros.

bioRxiv preprint doi: https://doi.org/10.1101/2022.02.24.481854; this version posted July 21, 2025. The copyright holder for this preprint (which was not certified by peer review) is the author/funder, who has granted bioRxiv a license to display the preprint in perpetuity. It is made available under aCC-BY 4.0 International license.

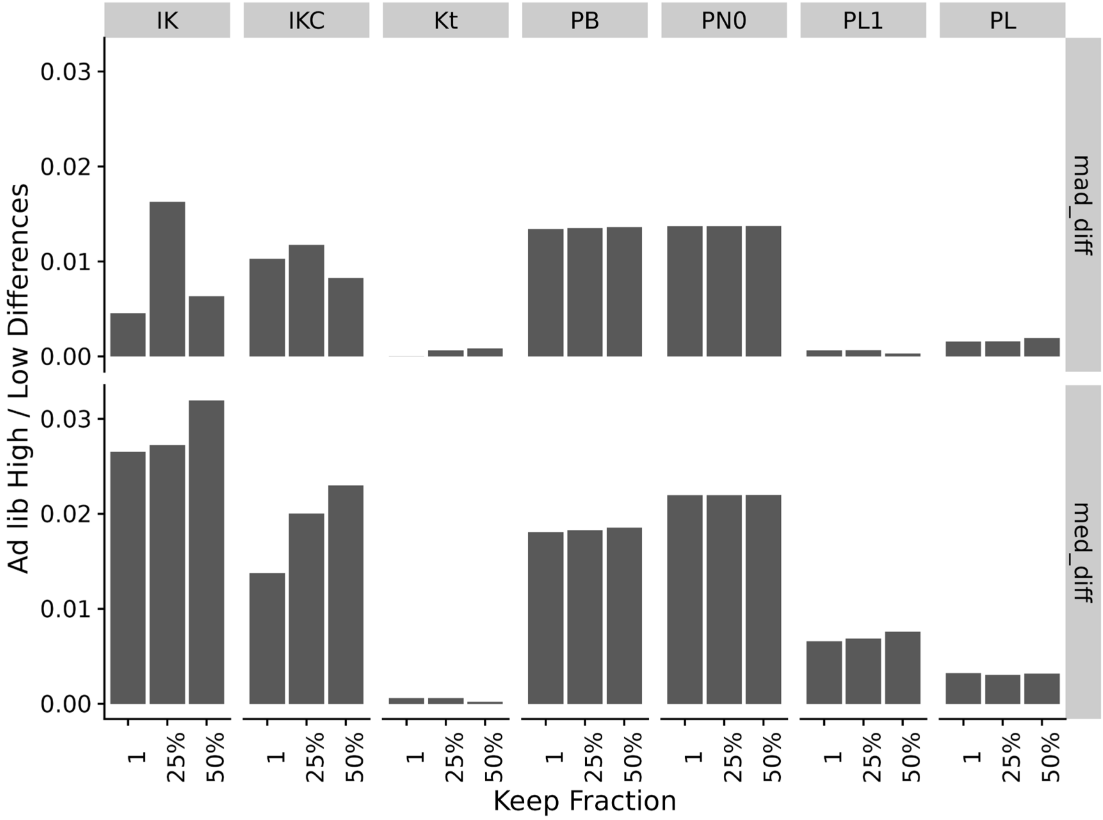

Figure S33. Difference of Ad lib feeding in high and low stamina medians and MADs by correlation method and applying different fractional cutoffs for rat stamina metabolomics data. Abbreviations for different measures and data are: IK: ICI-Kt; IKC: ICI-Kt * Completeness; Kt: Kendall-tau; PB: Pearson Base (raw values); PL: Pearson Log(x); PL1: Pearson Log(x + 1); PN0: Pearson No Zeros.

References
